# Systematic review: clinical characteristics of anti-N-methyl-D-aspartate receptor encephalitis

**DOI:** 10.3389/fnhum.2023.1261638

**Published:** 2023-11-20

**Authors:** Xi Zhao, Yuou Teng, Jingnian Ni, Ting Li, Jing Shi, Mingqing Wei

**Affiliations:** ^1^Dongzhimen Hospital, Beijing University of Chinese Medicine, Beijing, China; ^2^Department of Neurology, Dongzhimen Hospital, Beijing University of Chinese Medicine, Beijing, China

**Keywords:** anti-N-methyl-D-aspartate receptor encephalitis, anti-NMDAR, clinical characteristics, treatments, prognosis, systematic review

## Abstract

**Background:**

The number of reported cases of anti-N-methyl-D-aspartate receptor (anti-NMDAR) encephalitis has gradually increased since its discovery in 2007, while there are no uniform treatment guidelines.

**Objective:**

To summarize the clinical characteristics of patients with anti-NMDAR encephalitis and to analyze the factors affecting the disease prognosis.

**Methods:**

A systematic analysis of medical records was conducted, and PubMed, Embase, and Cochrane Library were searched from January 1, 2011, to December 31, 2021. Data were extracted, analyzed, and recorded in accordance with the Preferred Reporting Items for Systematic reviews and Meta-Analyses (PRISMA) guidelines.

**Results:**

This study included 472 case reports. Most patients had prodromal symptoms of about 2 weeks, including psychiatric symptoms (53.2%), flu-like symptoms (51.5%), and seizures (23.9%), among others. Poor prognoses were associated with patients who had autonomic instability (*p* = 0.010), central hypoventilation (*p* = 0.014), and ICU support (*p* = 0.002). Patients with a higher age of onset were more likely to develop central hypoventilation (OR 1.024, CI 1.006–1.042, *p* = 0.009), cognitive impairment (OR 1.023, CI 1.009–1.037, *p* = 0.001), and memory impairment (OR 1.034, CI 1.017–1.050, *p* < 0.001), whereas patients with a lower age were more likely to have seizures (OR 0.979, CI 0.965–0.993, *p* = 0.003). In this study, 97.0% of patients received immunotherapy, with the most commonly used treatment regimen being intravenous methylprednisolone (IVGC) and intravenous immunoglobulin (IVIG). When compared with other treatment regimens, the IVGC+IVIG regimen (*p* < 0.001) resulted in better prognoses.

**Conclusion:**

When encountering patients with fever, headache, and initial psychiatric symptoms of unknown etiology, clinicians should test their CSF for antibodies to distinguish autoimmune encephalitis. Patients with autonomic instability, central hypoventilation, and ICU support had poorer prognoses. Clinicians should be aware that older patients are more likely to develop central hypoventilation, cognitive impairment, and memory impairment, while younger patients are more likely to develop seizures. The IVGC+IVIG treatment regimen has better prognoses than others. This study includes case reports, which have obvious selection bias, and there are no unified standards to measure the severity of the disease. Therefore, in the future, larger samples and randomized controlled trials are needed to evaluate the efficacy of different treatment regimens.

## 1 Introduction

Anti-N-methyl-D-aspartate receptor (anti-NMDAR) encephalitis is an autoimmune encephalitis mediated by anti-NMDAR antibodies, first described by Dalmau et al. (2007). Autoimmune encephalitis refers to a class of encephalitis mediated by autoimmune mechanisms. Among them, anti-NMDAR encephalitis is the most common, accounting for approximately 54–80% of autoimmune encephalitis patients (Ren et al., 2021). The clinical features of anti-NMDAR encephalitis mainly include psychiatric and behavioral symptoms, seizures, cognitive dysfunction, and other signs (Liu et al., 2017), with autonomic dysfunction and central hypoventilation in some patients (Gable et al., 2012). Some patients may have detected presence of teratoma (Vitaliani et al., 2005), predominantly female patients aged 12–45 years old (Titulaer et al., 2013). Because of the variety of clinical manifestations of anti-NMDAR encephalitis, it is sometimes easy to misdiagnose it as other types of encephalitis, psychiatric disorders (Giné Servén et al., 2021; Subeh et al., 2021), or other diseases. The commonly used treatments include immunotherapy (IT), symptomatic treatment, supportive therapy, and rehabilitation therapy, among others (Dalmau et al., 2011; Titulaer et al., 2013; Sartori et al., 2015). A study including 577 patients (Titulaer et al., 2013) found complete recovery in about 50% of patients, with a median length of stay of 2.5 months, a recurrence rate of 12% at 2 years, and a mortality rate of 6%. Prevalence in children (<18 years old) is about 37–65% (Gable et al., 2012; Titulaer et al., 2013). One research (Gurrera, 2019) suggested that clinicians need to suspect the possibility of the presence of anti-NMDAR encephalitis if the child develops new behavioral symptoms in the midst of recent viral prodromes, or if they are accompanied by movement disorders, seizures, or insomnia.

There are no specific treatment guidelines for anti-NMDAR encephalitis, and most studies have been case reports. Therefore, this paper aims to conduct a systematic review analysis of case reports on anti-NMDAR encephalitis published in the past decade, to summarize the clinical characteristics and treatments of anti-NMDAR encephalitis, to analyze the factors that affect prognosis, and to provide theoretical support for clinicians in early diagnosis and reasonable treatments.

## 2 Methods

### 2.1 Search strategy

This systematic review was conducted according to the Preferred Reporting Items for Systematic reviews and Meta-Analyses (PRISMA) guidelines (Moher et al., 2015). This study conducted a comprehensive literature search on PubMed, Embase and Cochrane databases. The search term used is “Anti-N-Methyl-D-Aspartate Receptor Encephalitis” or “anti-nmda receptor encephalitis” or “anti-nmdar encephalitis”, and the search period was from 2011.1.1 to 2021.12.31.

### 2.2 Case selection

To obtain first-hand information on the cases and ensure the authenticity of the study, this research only selected case reports or case series that record individual patient characteristics, while excluding meta-analyses, prospective studies, abstract, questionnaires, commentaries, and other articles. Articles with incomplete case information (where the minimal requirement for inclusion was to record the patient’s age, gender, diagnosis, treatment, and prognosis) were also excluded. Based on criteria (Graus et al., 2016), the diagnosis of anti-NMDAR encephalitis requires the presence of the following three conditions: (A) One or more of the six major symptoms, including (Dalmau et al., 2007) psychiatric or cognitive abnormalities (Ren et al., 2021), language disorders (Liu et al., 2017), seizures (Gable et al., 2012), motor disturbances or involuntary movements (Vitaliani et al., 2005), decreased level of consciousness (Titulaer et al., 2013), autonomic dysfunction or central hypoventilation. (B) Positive anti-NMDAR antibody. (C) Reasonable exclusion of other causes. Each case is required to have a clear diagnosis of anti-NMDAR encephalitis without other central nervous system immune diseases, such as other types of encephalitis, multiple sclerosis (MS), neuromyelitis optica, etc. Considering the limitations of medication use in pregnant women, pregnant patients with anti-NMDAR encephalitis were excluded in this study. The study population was limited to the human range, excluding animal experiments and non-human case reports, and there were no restrictions on race, age or sex. The text is limited to English literature only, with no limitations on word count or the publication’s journal.

### 2.3 Data extraction and processing

For the 472 cases finally included, the full articles were carefully read, and available information was extracted. In order to uniformly describe the outcomes, patient prognosis was divided into four levels: “complete recovery,” “significant improvement,” “limited improvement” and “death.” If the patient was described as “completely recovered” or “almost completely recovered,” or was able to resume all activities, or the modified Rankin scale (mRS) score was determined to be 0 or 1, they were considered as “complete recovery.” If the patient was described as “I have improved” or “significantly improved,” or if there were some symptoms that still need improvement at the time of discharge, or if the mRS score was determined to be 2–4, they were considered as “significant improvement.” If the patient’s symptoms did not show significant improvement, or even worsen, or if the mRS score was determined to be 5 or 6, they were considered as “limited improvement.” If the patient died, they were considered as “death.”

### 2.4 Statistical analysis

In this study, IBM SPSS Statistics 26 was used for data processing. Chi-square test and logistic regression analysis were used to analyze the relationship between different symptoms, auxiliary examination results, treatment plans and prognosis.

## 3 Results

### 3.1 Search results

**Figure 1:**
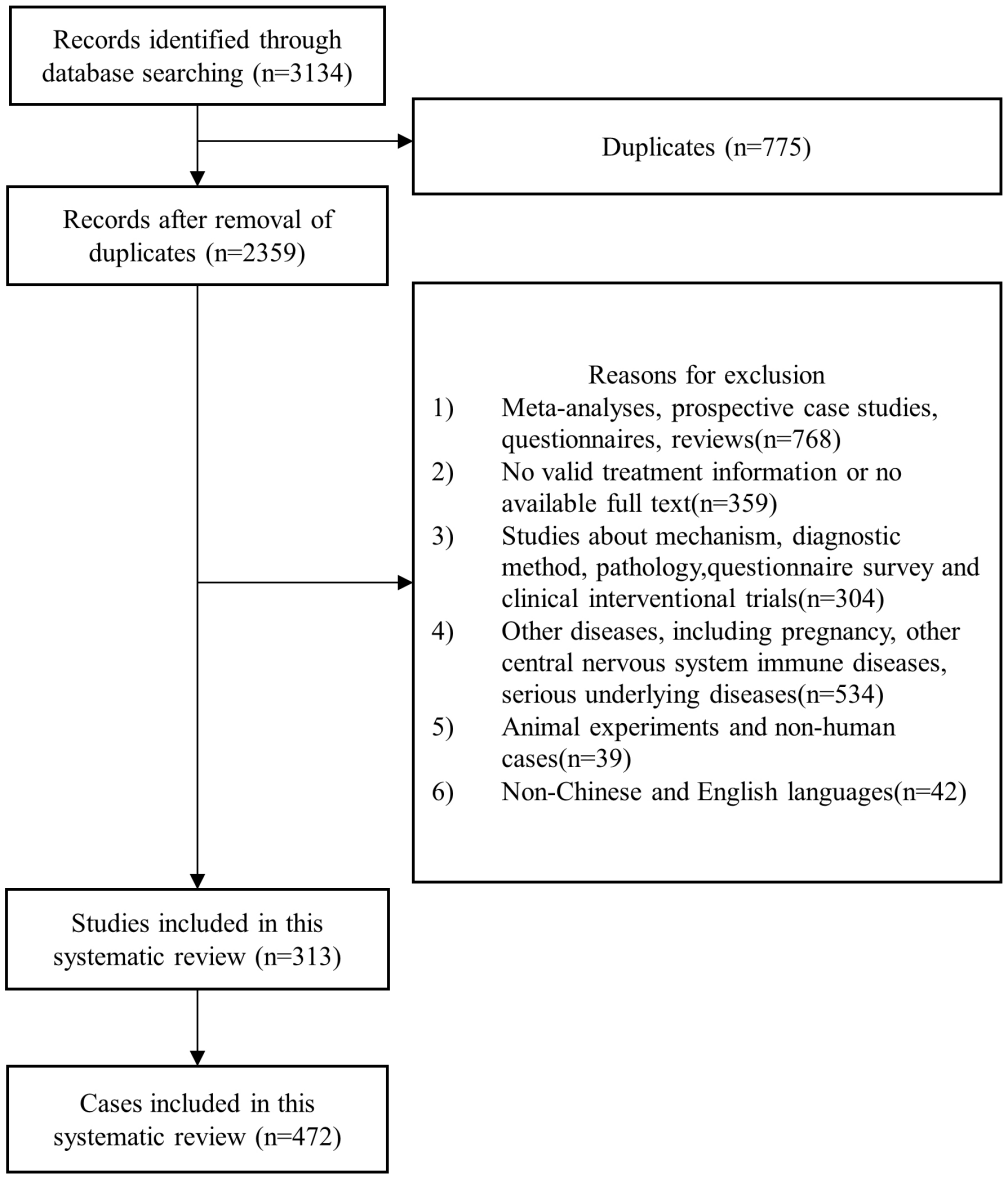
Flow chart of case selection.

Initial searches retrieved 3,134 articles, out of which 775 duplicate cases were removed. A total of 2,359 articles met the inclusion criteria, and finally 313 articles were included, with a total of 472 study cases. The flow chart of case selection is shown in Figure 1.

### 3.2 Demographic data

This study included a total of 472 confirmed cases of anti-NMDAR encephalitis, of which 77.8% (367/472) were female patients. The age of onset ranged from 0.8 to 84 years old, with a median onset age of 20 years old. More than half (239/472, 50.6%) of the patients had onset ages between 18 and 44 years old, and patients younger than 18 years old accounted for 42.8% (202/472) of cases. Among them, more than half (200/367, 54.5%) of female patients had onset ages between 18 and 44 years old, while more than half (54/105, 51.4%) of male patients had onset ages below 18 years old. Stratified analysis suggested that anti-NMDAR encephalitis was more common in females aged between 18 and 44 years old (*p* = 0.002). Ordered logistic regression analysis showed that gender did not significantly affect the prognosis of the disease (*p* = 0.991), but older age was associated with worse prognosis (*p* = 0.005).

Among all cases, the hospital stay ranged from 0.1 months to 68 months, with a median stay of 2 months. Of the 195 cases with follow-up records, the follow-up time ranged from 0.5 months to 60 months, with a median follow-up time of 8 months. In addition, 15/472 (3.2%) cases experienced relapse, with a relapse time range of 2 months to 20 months, and a median relapse time of 9 months. There were 21/472 (4.4%) patients who died, with causes of death including cardiovascular disease (6 cases), pulmonary disease (4 cases), infections (4 cases), organ failure (2 cases), and unknown causes (5 cases). Specific data information is shown in Table 1. The regression analysis of this study showed that the older the age of onset was correlated with the higher the likelihood of death (OR 0.959, CI 0.936–0.983, *p* = 0.001), while the younger the age of onset was correlated with the higher the likelihood of recurrence (OR 1.071, CI −1.133 ~ −1.012, *p* = 0.018). In addition, Patients with central hypoventilation (OR 0.335, CI 0.124–0.905, *p* = 0.031) or ICU support (OR 0.330, CI 0.135–0.805, *p* = 0.015) were more likely to have a fatal outcome. No other factors related to recurrence were found at this time.

**Table 1:** Demographic information.

Item (*n* = 472)	All patients (*n* = 472)	Age < 18 y (*n* = 202)	18 ≤ age < 45 (*n* = 239)	Age ≥ 45 (*n* = 31)
**Gender**				
Male	105 (22.2%)	54 (26.7%)	39 (16.3%)	12 (38.7%)
Female	367 (77.8%)	148 (73.3%)	200 (83.7%)	19 (61.3%)
**Prognosis**				
Complete recovery	165 (35.0%)	85 (42.1%)	72 (30.1%)	8 (25.8%)
Significant improvement	236 (50.0%)	87 (43.1%)	132 (65.3%)	17 (54.8%)
Limited improvement	50 (10.6%)	24 (11.9%)	24 (11.9%)	2 (6.4%)
Death	21 (4.4%)	6 (3.0%)	11 (4.6%)	4 (12.9%)
Recurrence	15 (3.2%)	9 (4.5%)	6 (2.5%)	0
**Tumor**	176/352 (50.0%)	43/136 (31.6%)	131/197 (66.5%)	2/19 (10.5%)
**Types**				
Ovarian teratoma	170/176 (96.6%)	42 (97.7%)	127 (96.9%)	1 (50.0%)
Mediastinal tumors	2 (1.1%)	1	1	
Uterine neuroendocrine differentiation carcinoma	1 (0.6%)			1
Testicular teratoma	1 (0.6%)		1	
Plasmacytoid cystic teratoma	1 (0.6%)		1	
Mucinous cystadenoma	1 (0.6%)		1	

### 3.3 Prodromal symptoms and disease symptoms

In this study, a total of 410/472 (86.9%) case reports described prodromal symptoms. Among them, more than half of the patients had psychiatric symptoms (218/410, 53.2%) or flu-like symptoms (211/410, 51.5%), as shown in Table 2. The duration of prodromal symptoms ranged from 1 day to 180 days, with 79.9% (191/239) of patients having prodromal symptoms lasting less than 2 weeks. The presence of prodromal symptoms did not show a significant relationship with the prognosis (*p* = 0.065), and different prodromal symptoms had no significant impact on the prognosis.

**Table 2:** Clinical symptom characteristics of anti-NMDAR encephalitis patients.

Symptom	Patients (*n* = 472)	*p* value^a^
Prodromal symptoms	410 (86.9%)	0.065
Flu-like symptoms	211/410 (51.5%)	0.564
Psychiatric symptoms	218/410 (53.2%)	0.070
Seizures	98/410 (23.9%)	0.919
Other symptoms^b^	29/410 (7.1%)	
Psychiatric symptoms	347 (73.5%)	0.200
Agitation	139/247 (56.3%)	0.361
Hallucination	131/247 (53.0%)	0.372
Anxiety	70/247 (28.3%)	0.804
Delusion	68/247 (27.5%)	0.711
Tension	50/247 (20.2%)	0.731
Depression	28/247 (11.3%)	0.726
Fear	11/247 (4.4%)	0.454
Other symptoms	100/247 (28.8%)	0.973
Seizures	320 (67.8%)	0.844
Movement disorders^c^	252 (53.4%)	0.423
Cognitive dysfunction	181 (38.3%)	0.387
Autonomic instability^d^	180 (38.1%)	0.010
Behavioral abnormalities	169 (35.8%)	0.187
Language disorders^e^	154 (32.6%)	0.352
Sleep disorders^f^	129 (27.3%)	0.285
Decreased consciousness	107 (22.7%)	0.874
Memory impairment	87 (18.4%)	0.494
Central hypoventilation	59 (12.5%)	0.014

a Ordered logistic regression analysis for various symptoms and prognosis.

b Such as limb motor dysfunction, gastrointestinal symptoms, infections, autonomic instability, etc.

c Such as orofacial dyskinesia, choreographic movements, abnormal gait, dystonic postures, etc.

d Such as tachycardia, bradycardia, excessive salivation, unstable blood pressure, urinary incontinence, etc.

e Such as mutism, dysarthria, aphasia, incomprehensible speech, etc.

f Such as insomnia, excessive sleepiness, abnormal behavior during sleep, etc.

The frequency of disease symptoms from high to low were as follows: psychiatric symptoms (agitation, hallucination, anxiety, delusion, tension, depression, fear), seizures, movement disorders, cognitive dysfunction, autonomic instability, behavioral abnormalities, language disorders, sleep disorders, decreased consciousness, memory impairment, central hypoventilation, with specific statistical data in Table 2. Regression analysis results showed that patients with autonomic instability (*p* = 0.010) and central hypoventilation (*p* = 0.014) had poorer prognosis, while other symptoms were not significantly correlated with prognosis.

Analyzing the relationship between age of onset and different symptoms, it was found that age of onset was positively correlated with central hypoventilation (OR 1.024, CI 1.006–1.042, *p* = 0.009), cognitive dysfunction (OR 1.023, CI 1.009–1.037, *p* = 0.001), and memory impairment (OR 1.034, CI 1.017–1.050, *p* < 0.001), but negatively correlated with seizures (OR 0.979, CI 0.965–0.993, *p* = 0.003).

### 3.4 Tumors

Tumors were recorded in 352 cases. 50.0% (176/352) of the patients had tumors, with ages ranging from 4 to 65 years old, with a median age of 21 years old. Only 2 out of the 352 cases were male, a mediastinal tumor (16 years old) and a testicular teratoma (30 years old). Female tumors mainly occurred between the ages of 13 to 36 years old (160/174, 92.0%). The most common tumor type was ovarian teratoma (170/176, 96.6%), while there were also two cases of mediastinal tumors, one case of bilateral uterine neuroendocrine differentiation carcinoma, one case of testicular teratoma, one case of plasmacytoid cystic teratoma, and one case of mucinous cystadenoma.

All patients with tumors underwent tumor resection surgery, and 6 patients also underwent prophylactic bilateral adnexectomy. Binary logistic regression analysis found that for every 1 year increase in age, the probability of having a tumor increased by 0.018 times (OR 1.018, CI 1.002–1.035, *p* = 0.030). The presence of a tumor did not show a significant correlation with the prognosis of the patient (*p* = 0.188). The relationship between different symptoms and the presence of a tumor was analyzed, and it was found that patients with seizures (OR 1.886, CI 1.144–3.110, *p* = 0.013) and memory impairment (OR 1.940, CI 1.049–3.588, *p* = 0.035) were more likely to have a tumor, while other symptoms were not related to tumors.

### 3.5 Intensive care unit

28.4% (134/472) of patients were admitted to the ICU. Compared with male patients, the probability of female patients requiring ICU support was higher (OR 2.077, CI 1.205–3.582, *p* = 0.009). Stratified analysis suggested that females aged from 18 to 44 years old had a higher probability of requiring ICU support (*p* = 0.024). Patients who required ICU support had a poorer prognosis (*p* = 0.002).

Except for gender, when symptoms such as decreased consciousness (OR 2.617, CI 1.596–4.290, *p* < 0.001), cognitive impairment (OR 1.771, CI 1.102–2.844, *p* = 0.018), central hypoventilation (OR 4.742, CI 2.562–8.776, *p* < 0.001), and agitation (OR 1.759, CI 1.088–2.842, *p* = 0.021) exist, patients were more likely to require ICU support. In terms of auxiliary examination, patients with elevated cerebrospinal fluid (CSF) cell count (OR 2.100, CI 1.224–3.604, *p* = 0.007) and extreme delta brush on electroencephalogram (EEG) (OR 4.411, CI 1.630–11.935, *p* = 0.003) were more likely to be admitted to ICU.

### 3.6 Auxiliary examinations

All cases had positive serum and/or CSF anti-NMDAR antibody tests. The chi-squared test showed a significant difference in the positive detection rates of serum and CSF antibodies (*p* = 0.014), indicating that the detection rate of positive antibodies in CSF was higher than that in serum.

Out of 472 patients, 401 cases reported the results of cranial magnetic resonance imaging (MRI). The results suggested that 63.8% (256/401) of the patients had normal results. One hundred and eight cases clearly described abnormalities in certain areas. Specific data is shown in Table 3. Patients with abnormal cranial MRI results tend to have poorer prognosis (*p* = 0.003). Further analysis showed that patients with temporal lobe abnormalities had a poorer prognosis (*p* = 0.047). Patients with abnormalities identified by MRI were more likely to develop symptoms of anxiety (OR 2.428, CI 1.241–4.751, *p* = 0.010). Patients with frontal lobe abnormalities were more likely to develop symptoms of depression (OR 3.302, CI 1.141–9.555, *p* = 0.028) and delusion (OR 2.480, CI 1.127–5.460, *p* = 0.024). Patients with marginal system abnormalities were more likely to develop symptoms of memory impairment (OR 2.581, CI 1.146–5.816, *p* = 0.022).

**Table 3:** Auxiliary examination results in patients.

Item	Patients	*p* value^a^
Anti-NMDAR antibody	472 (100.0%)	
Serum	268/284 (94.4%)	
CSF	412/421 (97.9%)	
Elevated CSF cell count	193/300 (64.3%)	0.294
EEG normal	45/336 (13.4%)	
EEG abnormal	291/336 (86.6%)	0.071
Seizures	55/291 (18.9%)	0.059
With extreme delta brush	34/291 (11.7%)	0.790
Other anomalies	202/291 (69.4%)	
Cranial MRI abnormal	145/401 (36.2%)	0.003
Temporal lobe abnormalities	48/108 (44.4%)	0.047
Frontal lobe abnormalities	36/108 (33.3%)	0.165
Depression	5/36 (13.9%)	0.028
Delusion	10/36 (27.8%)	0.024
Behavioral abnormalities	7/36 (19.4%)	0.034
Edge system abnormalities^b^	29/108 (26.9%)	0.146
Memory impairment	10/29 (34.5%)	0.022
Parietal abnormalities	11/108 (10.2%)	
Occipital lobe abnormalities	3/108 (2.8%)	

a Ordered logistic regression analysis of different positive results and different prognoses.

b It includes regions such as the hippocampus, thalamus, corpus callosum, insula, and so on.

### 3.7 Treatments

Among 472 patients, most (458/472, 97.0%) of them underwent IT, and specific data can be seen in Table 4. In addition to IT, some patients with epilepsy and psychiatric symptoms also received symptomatic drug therapy. In addition, 19/472 (4.0%) patients underwent electric convulsive therapy for the treatment of intractable epilepsy, and 10/472 (2.1%) patients received bortezomib treatment.

**Table 4:** Statistical table of treatment information.

Treatments^a^	Patients (*n* = 472)	*p* value^b^	Children (*n* = 202)	Adults (*n* = 270)
Without IT	14 (3.0%)	0.138	6 (3.0%)	8 (3.0%)
With IT	458 (97.0%)		196 (97.0%)	262 (97.0%)
IVGC	391 (82.8%)		163 (80.7%)	228 (84.4%)
IVIG	366 (77.5%)		167 (82.7%)	199 (73.7%)
PE	174 (36.9%)		58 (28.7%)	116 (43.0%)
2nd line IT	187 (39.6%)		87 (43.1%)	100 (37.0%)
RTX	144 (77.0%)		66 (75.9%)	78 (78.0%)
CTX	70 (37.4%)		32 (36.8%)	38 (38.0%)
MMF	16 (8.6%)		8 (9.2%)	8 (8.0%)
AZA	12 (6.4%)		5 (5.7%)	7 (7.0%)
**Combination of treatment options**				
Only IVGC	30 (6.4%)	0.182	9 (4.5%)	21 (7.8%)
Only IVIG	27 (5.7%)	0.134	13 (6.4%)	14 (5.2%)
Only PE	5 (1.1%)	0.219	1 (0.5%)	4 (1.5%)
Only 2nd line IT	5 (1.1%)	0.438	3 (1.5%)	2 (0.7%)
IVGC+IVIG	132 (28.0%)	<0.001	64 (31.7%)	68 (25.2%)
IVGC+PE	23 (4.9%)	0.061	4 (2.0%)	19 (7.0%)
IVGC+2nd line IT	10 (2.1%)	0.163	5 (2.5%)	5 (1.9%)
IVIG+PE	7 (1.5%)	0.817	1 (0.5%)	6 (2.2%)
IVIG+2nd line IT	10 (2.1%)	0.163	7 (3.5%)	3 (1.1%)
PE+2nd line IT	6 (1.3%)	0.594	4 (2.0%)	2 (0.7%)
IVGC+IVIG+PE	47 (10.0%)	0.835	17 (8.4%)	30 (11.1%)
IVGC+IVIG+2nd line IT	70 (14.8%)	0.212	37 (18.3%)	33 (12.2%)
IVGC+PE+2nd line IT	13 (2.8%)	0.514	3 (1.5%)	10 (3.7%)
IVIG+PE+2nd line IT	7 (1.5%)	0.717	4 (2.0%)	3 (1.1%)
IVGC+IVIG+PE+2nd line IT	66 (14.0%)	0.226	24 (11.9%)	42 (15.6%)

a IVGC refers to intravenous injection of glucocorticoids, IVIG refers to intravenous injection of immunoglobulin, PE refers to plasma exchange, 2nd line IT refers to second-line immunotherapy, IT refers to immunotherapy, RTX refers to rituximab, CTX refers to cyclophosphamide, MMF refers to mycophenolate and AZA refers to azathioprine.

b Ordered logistic regression analysis of different treatment regimens and prognosis.

In different combinations of treatments, irrespective of children or adults, the most commonly used treatment was intravenous glucocorticoids (IVGC) + intravenous immune globulin (IVIG). The use of IT did not significantly affect the prognosis (*p* = 0.138). Compared with adults, children used more IVIG and less plasmapheresis (PE) (*p* = 0.032). Patients who received IVGC+IVIG had a better prognosis compared with other treatments (*p* < 0.001). Regression analysis suggested that there was little difference in the impact of each treatment on prognosis in children, while adults had a better prognosis when treated with IVGC+IVIG (*p* = 0.001).

187/472 (39.6%) patients received second-line immunotherapy (2nd line IT), with the most commonly used being rituximab (RTX) alone. The specific data are shown in Table 5. Ordered logistic regression analysis suggested that different 2nd line IT regimens had no significant impact on prognosis.

**Table 5:** Statistical table of 2nd line IT.

Treatments^a^	Patients (*n* = 187)	*p* value^b^
RTX	93 (49.7%)	0.562
RTX + CTX	47 (25.1%)	0.329
CTX	19 (10.2%)	0.914
MMF	14 (7.5%)	0.810
AZA	8 (4.3%)	0.238
RTX + CTX + AZA	3 (1.6%)	
RTX + MMF	2 (1.1%)	
CTX + AZA	1 (0.5%)	

a All drug combination regimens mentioned in the literature.

b Ordinal logistic regression analysis of different second-line drug treatment regimens and prognosis.

## 4 Discussion

This article systematically analyzes the clinical characteristics and treatment effects of anti-NMDAR encephalitis. A total of 472 cases diagnosed with anti-NMDAR encephalitis were included in this study, with female patients accounting for 77.8% and a median onset age of 20 years old, which is basically the same as the previous study (Titulaer et al., 2013). Consistent with the views of Dalmau et al. (2011), patients with anti-NMDAR encephalitis usually have Prodromal symptoms of about 2 weeks, including psychiatric symptoms, epilepsy, headache, fever, nausea, vomiting, and other symptoms. This reminds clinicians that when encountering patients with such symptoms, they should consider the possibility of anti-NMDAR encephalitis and timely conduct antibody testing and targeted treatment. In addition to non-specific viral-like symptoms, many patients seek treatment from psychiatric departments due to psychiatric symptoms, and even some patients are misdiagnosed with mental illnesses, delaying treatment. A clinical observation on anti-NMDAR encephalitis (Irani et al., 2010) showed that 77% (34/44) of patients with early clinical features of psychosis. Considering the high similarity between anti-NMDAR encephalitis and some symptoms of psychosis (hallucinations, delusions, agitation, etc.), Subeh et al. (2021) suggested that in cases where psychosis was a prodromal symptom, even if there were no other neurological manifestations, anti-NMDAR encephalitis should be considered. Another study (Giné Servén et al., 2021) also suggested that patients with cycloid psychosis phenotype presenting with the first episode of psychosis should undergo CSF analysis to exclude the possibility of autoimmune encephalitis. Therefore, it is recommended in this study that patients who seek medical attention for the first time with psychiatric symptoms (hallucinations, delusions, agitation, etc.) should be examined for autoimmune encephalitis, and improve CSF antibody testing to reduce the occurrence of misdiagnosis.

Patients with anti-NMDAR encephalitis may exhibit movement disorders, including orofacial dyskinesia, choreiform movements, abnormal gait, and dystonic postures. The clinical manifestations of anti-Homer-3 antibody encephalitis are subacute or insidious attacks of cerebellar ataxia (Liu et al., 2021). There have been fewer case reports of anti-Homer-3 antibody encephalitis, mostly with adult onset, and the youngest age of onset recorded was 10 years old (Kuang et al., 2022), a Chinese boy. He presented with inattention, irritability, and slurred speech. Neurologic examination revealed dysarthria and nystagmus, and bipedal gait showed marked ataxia. Positive anti-Homer-3 antibodies were detected in CSF but not in serum. Clinicians encountered patients with movement disorders need to consider the possibility of autoimmune mediation, as well as identify different types of encephalitis. In addition, anti-glutamic acid decarboxylase-65 (GAD65), an enzyme that converts excitatory glutamate to inhibitory gamma-aminobutyric acid (GABA), can lead to a range of disorders, such as tonic syndrome, cerebellar ataxia, epilepsy, limbic encephalitis, and meningoencephalitis (Muñoz-Lopetegi et al., 2020; Kuang et al., 2023). Its clinical presentation has many similarities with anti-NMDAR encephalitis and also requires careful differentiation.

This study found that patients with autonomic instability, central hypoventilation and the need for ICU support have a poor prognosis. Additionally, elderly patients with central hypoventilation or a history of ICU admission are more likely to have fatal outcomes. Wang and Xiao (2020) pointed out in a review of the prognosis of anti-NMDAR encephalitis that autonomic dysfunction during the course of the disease may lead to severe complications (including arrhythmia and cardiac arrest) and poor prognosis. Severe autonomic dysfunction and central hypoventilation usually require ICU support, and ICU support is usually an independent risk factor for death. Titulaer et al. (2013) reported that not admitted to ICU is one of the prognostic factors for good prognosis. This study found that women, especially middle-aged women, are more likely to require ICU support. In addition to gender, patients with symptoms of decreased consciousness level, cognitive impairment, central hypoventilation, and agitation are also highly likely to require ICU support. Additionally, patients who have high CSF cell counts and exhibit extreme delta brush on EEG are also more likely to enter the ICU. This reminds clinicians to pay special attention to such patients and intervene early.

This study found that older age at the onset of anti-NMDAR encephalitis was associated with a greater likelihood of central hypoventilation, cognitive impairment, and memory impairment, while younger age at onset was associated with a greater likelihood of seizures. Titulaer et al. (2013) also reported that compared with adults, children are more likely to have seizures or movement disorders, while memory impairment and central hypoventilation are more common among adults. A systematic review by Zhang et al. (2017) reported that the proportion of children with initial presentations of seizures and psychiatric symptoms is relatively higher. This reminds clinicians that the focus needs to change when treating patients of different ages. For example, younger patients should be monitored for seizures, while older patients should be monitored for central hypoventilation, cognitive impairment, and memory impairment, and receive targeted treatment in a timely manner to avoid affecting their prognosis.

In this study, 50.0% of patients had a history of tumors, which is relatively higher than in previous studies (Kayser and Dalmau, 2016; Zhang et al., 2017). The reasons are as follows: Dalmau et al. (2007) with the gradual increase in awareness of anti-NMDAR encephalitis, primary care physicians are now consciously screening for tumors, compared with in the past. At the same time, examination technology is also constantly developing, which further increases the detection rate (Ren et al., 2021). In order to obtain first-hand medical records, this study selected individual case reports. The authors may have deliberately selected cases with tumors in order to increase the reference value of the article, which unwittingly increased the exposure. Therefore, there is a selection bias in the cases of this study.

The majority of tumors are ovarian teratomas, and tumors in female patients mainly occur between the ages of 11 and 37 years old (94.3%), which is slightly different from the results of Titulaer et al.’s study (Titulaer et al., 2013) of 12–44 years old, considering the selection bias of the case reports. Analysis found that as age increases, the probability of tumors also gradually increases. However, whether the presence of tumors is related to the prognosis of patients has no significant correlation, and similar conclusions have been drawn in previous studies (Thomas et al., 2013; Titulaer et al., 2013). This study found that patients who develop seizures and memory impairment are more likely to have tumors. Currently, there is no research proposing the same theory, and it is considered that the reliability of this viewpoint still needs to be verified by a larger and more rigorous case analysis due to the selection bias of the cases in this study. In this study, all patients with tumors underwent tumor resection surgery, and six patients also underwent Prophylactic bilateral salpingo-oophorectomy. An interesting report (Masghati et al., 2014) describes the case of twin sisters who were previously healthy. Both were diagnosed with anti-NMDAR encephalitis at the age of 27, and no tumors were detected in either case. After similar treatments, the older sister died of arrhythmia, while the younger sister almost fully recovered after undergoing prophylactic bilateral salpingo-oophorectomy. Perhaps other factors contributed to the drastically different outcomes of the sisters, but this does provide clinicians with a new idea - that female patients could potentially avoid malignant events by undergoing prophylactic bilateral salpingo-oophorectomy. Of course, there are many issues to consider, such as ethical considerations, patient and family willingness, and further etiological studies and clinical observations are needed in the future to draw conclusions.

Most patients with anti-NMDAR encephalitis undergo cranial MRI examinations. In this study, 63.8% of the cases were described as normal, which is consistent with the conclusions of other studies (Dalmau et al., 2008; Gable et al., 2012). The most common abnormal site described in cranial MRI was the temporal lobe, and patients with temporal lobe abnormalities had a worse prognosis. However, several studies have found no significant correlation between MRI abnormalities and poor prognosis (Dalmau et al., 2008; Irani et al., 2010; Wang et al., 2016; Chi et al., 2017; Montmollin et al., 2017). It is undeniable that our study has selection bias, varying quality of case reports, and questionable reliability of the data. Other studies are also limited by the sample size (Dalmau et al., 2008; Irani et al., 2010), geographic and racial factors (Wang et al., 2016; Chi et al., 2017), and special patient populations (Montmollin et al., 2017), so their conclusions are limited. In addition, the MRI results may vary depending on the stage of the disease, which can also affect the accuracy of the analysis. Future studies on the relationship between cranial MRI and prognosis in patients with anti-NMDAR encephalitis could consider increasing the sample size and overcoming geographic and racial limitations; they could also differentiate different stages of the disease for analysis of cranial MRI.

In this study, 97.0% of the patients received IT, with the most commonly used treatment being IVGC+IVIG, which is consistent with the findings of Titulaer et al. (2013). This study found that there was no correlation between the use of IT and disease prognosis. In contrast, another larger study (Broadley et al., 2019) found that not using IT was a variable associated with poor prognosis of anti-NMDAR encephalitis. Considering that there is no unified standard to measure the severity of the disease in case reports, we cannot determine if patients who did not receive IT had milder symptoms, which compromised the accuracy of evaluating the efficacy of IT in this study. In contrast, more studies (Gabilondo et al., 2011; Titulaer et al., 2013; Balu et al., 2019) have suggested that the early initiation of IT is critical for achieving good outcomes. The results of this study suggest that the IVGC+IVIG treatment regimen can lead to better prognosis compared to other treatment regimens. When analyzing the effect of different treatment regimens for children and adults, it was found that adults had better prognosis with the use of IVGC+IVIG; perhaps due to the small number of cases, there was not a large difference in prognosis among different treatment regimens for children. This study analyzed information extracted from a large number of real cases, and although there was selection bias, the research results are still of reference value. IVGC+IVIG may cause side effects such as increased susceptibility to infection, elevated blood pressure, allergic reactions, gastrointestinal abnormalities, endocrine system abnormalities, headaches, and palpitations. However, these side effects are acceptable when compared to the severe prognosis of anti-NMDAR encephalitis. Therefore, this study recommends the use of IVGC+IVIG treatment regimen for anti-NMDAR encephalitis patients, regardless of whether they are children or adults.

In this study, 4.4% of the patients died and 3.2% experienced recurrence. The mortality and recurrence rates were generally lower than in other studies, possibly due to selection bias in case reports. The median follow-up time in this study was 8 months, while many studies (Dalmau et al., 2008; Irani et al., 2010; Gabilondo et al., 2011) have demonstrated that the median interval to recurrence is 2 years, suggesting that some cases in this study had a relatively short follow-up time, which failed to record the recurrence of some patients. This study found that the younger the age, the higher the likelihood of recurrence. However, given the limited number of recurrent cases included in this study, this view needs further careful consideration. In addition, some studies (Titulaer et al., 2013; Staley et al., 2019) have suggested that the risk of recurrence is higher in patients who did not receive timely immunotherapy during the first onset or in patients without potential tumors.

## 5 Conclusion

Since this study included mostly case reports, there was obvious selection bias and no unified standard to measure the severity of the disease. Therefore, future studies with larger sample sizes and randomized controlled trials are needed to assess the efficacy of different treatment options. This study suggests that when examining patients with fever or headache of unknown causes or patients with first-time manifestations of mental symptoms, clinical doctors should test for anti-NMDAR encephalitis antibodies to distinguish autoimmune encephalitis. The analysis found that patients with autonomic instability, central hypoventilation, and ICU support had poorer prognoses. Furthermore, young and middle-aged female patients with decreased consciousness, cognitive impairment, central hypoventilation, agitation symptoms, increased CSF cell counts, and extreme delta brushing in EEG are more likely to be admitted to the ICU, and these patients should be a focus of clinical attention. In addition, the older the age of onset in anti-NMDAR encephalitis patients, the more likely they are to develop central hypoventilation, cognitive impairment, and memory impairment, while the younger, the more likely they are to have seizures. In terms of treatment, the most widely chosen treatment option currently is IVGC+IVIG, which is also considered by this study as having a better prognosis.

## Data availability statement

The original contributions presented in the study are included in the article/Supplementary material, further inquiries can be directed to the corresponding author.

## Author contributions

XZ: Data curation, Formal analysis, Investigation, Resources, Writing – original draft. YT: Conceptualization, Writing – review & editing. JN: Writing – review & editing. TL: Writing – review & editing. JS: Supervision, Writing – review & editing. MW: Project administration, Supervision, Writing – review & editing.
