# Supplementary material for: Systematic review: clinical characteristics of anti-N-methyl-D-aspartate receptor encephalitis
Source: Front Hum Neurosci. 2023 Nov 20;17:1261638. doi: 10.3389/fnhum.2023.1261638 (PMC10694196; doi:10.3389/fnhum.2023.1261638)
Supplement: Supplementary file 1 [file Table_1.DOCX]

References of included studies(1-313)

1. Abdul Rashid AM, Mohamad Mokhtar E, Md Noh MSF. A case of anti-N-methyl-d-aspartate receptor encephalitis. SAGE Open Med Case Rep. 2020;8:2050313x20926431.

2. Abdul-Rahman ZM, Panegyres PK, Roeck M, Hawkins D, Bharath J, Grolman P, et al. Anti-N-methyl-D-aspartate receptor encephalitis with an imaging-invisible ovarian teratoma: a case report. J Med Case Rep. 2016;10(1):296.

3. Abou Khaled KJ, Azar CE, Haidar MB. Ictal and interictal FDG-PET in anti-NMDAR encephalitis with mutism. Radiol Case Rep. 2021;16(12):3892-7.

4. Abuzaid M, Alomar O, Salem H. Paraneoplastic Teratoma-associated Anti-N-Methyl-D-Aspartate Receptor Encephalitis: The First Published Report from Saudi Arabia. Cureus. 2018;10(10):e3527.

5. Akiyama H, Sasaki R, Hasegawa Y. Efficacy of perampanel for anti-N-methyl-D-aspartate receptor encephalitis: A case report. Medicine (Baltimore). 2019;98(2):e14033.

6. Alkayali T, Bell S, Bass C, Lattanzio N, Kraitman N. Not Your Average Seizure: A Case of N-Methyl-D-Aspartate Receptor Encephalitis and Review of Literature. Cureus. 2020;12(7):e9068.

7. Alqassmi AA, Alaklabi F, Alzomor OA, Salih MA. The challenge of diagnosing and successfully treating anti-NMDA receptor encephalitis in a toddler. Sudan J Paediatr. 2021;21(1):76-81.

8. Amugoda C, Chini Foroush N, Akhlaghi H. Anti-NMDAR Encephalitis: Higher Suspicious Needed for Earlier Diagnosis (Case Report, Literature Review and Diagnostic Criteria). Case Rep Neurol Med. 2019;2019:7476254.

9. Appu M, Noetzel M. Clinically significant response to zolpidem in disorders of consciousness secondary to anti-N-methyl-D-aspartate receptor encephalitis in a teenager: a case report. Pediatr Neurol. 2014;50(3):262-4.

10. Ashida S, Tanaka E, Ogura S, Maezono K, Nagakane Y. Case of an anti-N-methyl-d-aspartate receptor encephalitis patient responding to cyclophosphamide with significant brain volume recovery. Clinical and Experimental Neuroimmunology. 2020;11(4):238-41.

11. Azizyan A, Albrektson JR, Maya MM, Pressman BD, Moser F. Anti-NMDA encephalitis: an uncommon, autoimmune mediated form of encephalitis. J Radiol Case Rep. 2014;8(8):1-6.

12. Babbitt CJ, Lake J, Levine GK. Use of dexmedetomidine as adjunctive therapy for anti-N-methyl-D-aspartate receptor encephalitis. J Pediatr Intensive Care. 2013;2(3):143-5.

13. Baker J, Jeziorkowski C, Siebe C, Osborn MB. Two Cases of Anti-NMDA Receptor Encephalitis. West J Emerg Med. 2016;17(5):623-6.

14. Bashiri FA, Al-Rasheed AA, Hassan SM, Hamad MHA, El Khashab HY, Kentab AY, et al. Auto-immune anti-N-methyl-D-aspartate receptor (anti-NMDAR) encephalitis: three case reports. Paediatr Int Child Health. 2017;37(3):222-6.

15. Begum J, Aziz Z, Sahoo SK, Majumder R, Sable MN. Anti-N-Methyl-D-Aspartate Receptor Encephalitis Associated with Mature Ovarian Teratoma in a Young Adolescent: A Case Report. J Pediatr Adolesc Gynecol. 2021.

16. Behrendt V, Krogias C, Reinacher-Schick A, Gold R, Kleiter I. Bortezomib Treatment for Patients With Anti-N-Methyl-d-Aspartate Receptor Encephalitis. JAMA Neurol. 2016;73(10):1251-3.

17. Berg A, Byrne R, Coffey BJ. Neuroleptic Malignant Syndrome in a Boy with NMDA Receptor Encephalitis. J Child Adolesc Psychopharmacol. 2015;25(4):368-71.

18. Boeck AL, Logemann F, Krauß T, Hussein K, Bültmann E, Trebst C, et al. Ovarectomy despite Negative Imaging in Anti-NMDA Receptor Encephalitis: Effective Even Late. Case Rep Neurol Med. 2013;2013:843192.

19. Bradley L. Rehabilitation following anti-NMDA encephalitis. Brain Inj. 2015;29(6):785-8.

20. Brenton JN, Kim J, Schwartz RH. Approach to the Management of Pediatric-Onset Anti-N-Methyl-d-Aspartate (Anti-NMDA) Receptor Encephalitis: A Case Series. J Child Neurol. 2016;31(9):1150-5.

21. Bu Y, Zhang T, Guo J. Case report: anti-N-Methyl-D-Aspartate receptor encephalitis and bilateral temporal calcifications. BMC Neurol. 2020;20(1):386.

22. Buechner S, Florio I, Sixt GJ, Teatini F. A critical reflection on our first patient presenting with Anti-Nmethyl- D-aspartate receptor encephalitis. Neurol Int. 2019;11(3):8253.

23. Byrne S, McCoy B, Lynch B, Webb D, King MD. Does early treatment improve outcomes in N-methyl-D-aspartate receptor encephalitis? Dev Med Child Neurol. 2014;56(8):794-6.

24. Camacho A, Núñez N, Armangué T, Simón R. Myorhythmia-Like Dyskinesia Affecting the Face and Ear Associated With Anti-N-Methyl-d-Aspartate Receptor Encephalitis. Mov Disord Clin Pract. 2016;3(4):425-6.

25. Cantalupo G, Fasano A. Pediatric freezing of gait caused by anti-NMDAR encephalitis. Mov Disord. 2019;34(5):756-7.

26. Cantarín-Extremera V, Duat-Rodríguez A, González-Gutiérrez-Solana L, López-Marín L, Armangue T. Clinical case of anti-N-methyl-D-aspartate receptor encephalitis in an 8-month-old patient with hyperkinetic movement disorder. Pediatr Neurol. 2013;48(5):400-2.

27. Casares M, Skinner HJ, Gireesh ED, Wombles C, Schweitzer J, Gwyn PG, et al. Successful Intrathecal Rituximab Administration in Refractory Nonteratoma Anti-N-Methyl-D-Aspartate Receptor Encephalitis: A Case Report. J Neurosci Nurs. 2019;51(4):194-7.

28. Chakrabarty B, Tripathi M, Gulati S, Yoganathan S, Pandit AK, Sinha A, et al. Pediatric anti-N-methyl-D-aspartate (NMDA) receptor encephalitis: experience of a tertiary care teaching center from north India. J Child Neurol. 2014;29(11):1453-9.

29. Chan J, Gollapudi A, Greenberg S, Goldin A, Debiec KE. Case Report: Bilateral Teratomas with Immature Focus in a Patient Presenting with Immune Mediated Encephalitis. Journal of Pediatric and Adolescent Gynecology. 2019;32(2):229-30.

30. Chang HS, Hu LY, Wen CH, Su N. Anti-N-methyl-D-aspartate receptor encephalitis with isolated regressive behaviour: A case report. Hong Kong Journal of Emergency Medicine. 2016;23(4):242-5.

31. Chen B, Wang Y, Geng Y, Huang Y, Guo S, Mao X. Marked improvement of anti-N-methyl-D-aspartate receptor encephalitis by large-dose methylprednisolone and plasmapheresis therapy combined with (18)F-fluorodeoxyglucose positron emission tomography imaging: A case report. Exp Ther Med. 2014;8(4):1167-9.

32. Chen YM, Kuo PH. Extreme delta brush patterns guide the complex motor phenomenon of anti-NMDA receptor encephalitis: A case report. Medicine (Baltimore). 2020;99(9):e19384.

33. Cheng H, Yang F, Zhang J, Xu L, Jia L, Zhao D, et al. Case Report: Anti-NMDA Receptor Encephalitis With Bilateral Hearing Loss as the Initial Symptom. Front Neurol. 2021;12:648911.

34. Chheda A, Desai KM, Joshi R, Thakkar M, Pillai R, Walzade P, et al. A case of anti-NMDAR encephalitis with peculiar gyratory events that reignites the epilepsy versus movement disorders debate. Seizure. 2020;83:193-6.

35. Chiu HC, Su YC, Huang SC, Chiang HL, Huang PS. Anti-NMDAR encephalitis with ovarian teratomas: Review of the literature and two case reports. Taiwan J Obstet Gynecol. 2019;58(3):313-7.

36. Cho EH, Byun JM, Park HY, Kang MR, Jung YJ, Jeong CH, et al. The first case of Anti-N-methyl-d-aspartate receptor encephalitis (Anti-NMDAR encephalitis) associated with ovarian mucinous cystadenoma: A case report. Taiwan J Obstet Gynecol. 2019;58(4):557-9.

37. Choe CU, Karamatskos E, Schattling B, Leypoldt F, Liuzzi G, Gerloff C, et al. A clinical and neurobiological case of IgM NMDA receptor antibody associated encephalitis mimicking bipolar disorder. Psychiatry Research. 2013;208(2):194-6.

38. Choreño-Parra JA, de la Rosa-Arredondo T, Garibay-Gracián A, Montes de Oca-Vargas I, Capi-Casillas D, González-Pérez KV, et al. Atypical Association of Autoimmune Limbic Encephalitis with Anti-NMDA Receptor Antibodies in a Young Male Patient: Clinical, Imaging, and Neuropsychological Characteristics. Case Rep Neurol. 2021;13(2):541-8.

39. Chua GT, Zhou D, Ho ACC, Chan SHS, Yu CY, Lau YL. A case report of complement C4B deficiency in a patient with steroid and IVIG-refractory anti-NMDA receptor encephalitis. BMC Neurol. 2020;20(1):339.

40. Cleland N, Lieblich S, Schalling M, Rahm C. A 16-year-old girl with anti-NMDA-receptor encephalitis and family history of psychotic disorders. Acta Neuropsychiatr. 2015;27(6):375-9.

41. Cleverly K, Gambadauro P, Navaratnarajah R. Paraneoplastic anti-N-methyl-D-aspartate receptor encephalitis: have you checked the ovaries? Acta Obstet Gynecol Scand. 2014;93(7):712-5.

42. Cohen AL, Wong-Kisiel LC. Case of a two-year-old boy with recurrent seizures, abnormal movements, and central hypoventilation. Semin Pediatr Neurol. 2014;21(2):114-8.

43. Colley S, Smith J. Sore eyes and psychosis. BMJ Case Rep. 2014;2014.

44. Consoli A, Ronen K, An-Gourfinkel I, Barbeau M, Marra D, Costedoat-Chalumeau N, et al. Malignant catatonia due to anti-NMDA-receptor encephalitis in a 17-year-old girl: case report. Child Adolesc Psychiatry Ment Health. 2011;5(1):15.

45. Constantinides VC, Kasselimis DS, Paraskevas GP, Zacharopoulou M, Andreadou E, Evangelopoulos ME, et al. Anti-NMDA receptor encephalitis presenting as isolated aphasia in an adult. Neurocase. 2018;24(4):188-94.

46. Cooper JJ, Afzal KI. Safety of Electroconvulsive Therapy in 2 Very Young Pediatric Patients With Catatonia Related to Anti-N-methyl-D-aspartate Receptor Encephalitis. J ect. 2019;35(3):216-7.

47. Crimì F, Camporese G, Lacognata C, Fanelli G, Cecchin D, Zoccarato M. Ovarian Teratoma or Uterine Malformation? PET/MRI as a Novel Useful Tool in NMDAR Encephalitis. In Vivo. 2018;32(5):1231-3.

48. Curnow SR, Chow S, Yiu EM, Kornberg AJ. Anti-N-Methyl-d-Aspartate Receptor Encephalitis Presenting as Isolated Psychosis in an Adolescent Female. Pediatr Neurol. 2019;97:76-7.

49. Dabner M, McCluggage WG, Bundell C, Carr A, Leung Y, Sharma R, et al. Ovarian teratoma associated with anti-N-methyl D-aspartate receptor encephalitis: a report of 5 cases documenting prominent intratumoral lymphoid infiltrates. Int J Gynecol Pathol. 2012;31(5):429-37.

50. Day GS, High SM, Cot B, Tang-Wai DF. Anti-NMDA-receptor encephalitis: case report and literature review of an under-recognized condition. J Gen Intern Med. 2011;26(7):811-6.

51. De Maeseneire C, Tahry RE, Santos SF. A case of anti-NMDA receptor encephalitis revealed by insular epilepsy. Epileptic Disord. 2017;19(4):471-5.

52. Deiva K, Pera MC, Maurey H, Chrétien P, Archambaud F, Bouilleret V, et al. Sudden and isolated Broca's aphasia: a new clinical phenotype of anti NMDA receptor antibodies encephalitis in children. Eur J Paediatr Neurol. 2014;18(6):790-2.

53. Delangle R, Demeret S, Canlorbe G, Chelon L, Belghiti J, Gonthier C, et al. Anti-NMDA receptor encephalitis associated with ovarian tumor: the gynecologist point of view. Arch Gynecol Obstet. 2020;302(2):315-20.

54. Dericioglu N, Vural A, Acar P, Agayeva N, Ismailova V, Kurne A, et al. Antiepileptic treatment for anti-NMDA receptor encephalitis: the need for video-EEG monitoring. Epileptic Disord. 2013;15(2):166-70.

55. Di Capua D, García-Ptacek S, García-García ME, Abarrategui B, Porta-Etessam J, García-Morales I. Extreme delta brush in a patient with anti-NMDAR encephalitis. Epileptic Disord. 2013;15(4):461-4.

56. Di Luca DG, Margolesky JH. Severe tooth loss secondary to orofacial dyskinesias in anti-NMDA receptor encephalitis. BMJ Case Rep. 2019;12(3).

57. Dizon AM, Samples KL, Kimball KJ, Kilgore LC. Paraneoplastic anti-n-methyl-d-apartate-receptor encephalitis associated with an immature teratoma. Clinical Ovarian and other Gynecologic Cancer. 2012;5(2):97-8.

58. do Valle DA, Santos M, Zeny MS, Cordeiro ML. Pediatric Anti-N-methyl-D-aspartate Receptor Encephalitis Mimicking Glutaric Aciduria Type 1: A Case Report. Front Neurol. 2020;11:587324.

59. Doden T, Sekijima Y, Ikeda J, Ozawa K, Ohashi N, Kodaira M, et al. Postpartum Anti-N-methyl-D-aspartate Receptor Encephalitis: A Case Report and Literature Review. Intern Med. 2017;56(3):357-62.

60. Dubey S, Ghosh R, Dubey MJ, Sengupta S, Benito-León J, Ray BK. Bilateral thalamic changes in anti-NMDAR encephalitis presenting with hemichorea and dystonia and acute transient psychotic disorder. J Neuroimmunol. 2020;347:577329.

61. Dulcey I, Céspedes MU, Ballesteros JL, Preda O, Aneiros-Fernández J, Clavero PA, et al. Necrotic mature ovarian teratoma associated with anti-N-methyl-D-aspartate receptor encephalitis. Pathol Res Pract. 2012;208(8):497-500.

62. El Hanna J, Quenum C, Arsalane A. Anti-NMDAR encephalitis in a 19 year old female patient with ovarian teratoma: A case report. Eur J Obstet Gynecol Reprod Biol X. 2021;11:100129.

63. Elamin M, Mir FF, Alam Ali DM, Awad M, Mohammed Alshamy M, Ahmad K. Psychiatric Manifestation of Anti-NMDA Receptor Autoimmune Encephalitis. Dubai Medical Journal. 2019;1(1-4):26-8.

64. Endres D, Perlov E, Stich O, Rauer S, Maier S, Waldkircher Z, et al. Hypoglutamatergic state is associated with reduced cerebral glucose metabolism in anti-NMDA receptor encephalitis: A case report. BMC Psychiatry. 2015;15(1).

65. Endres D, Rauer S, Kern W, Venhoff N, Maier SJ, Runge K, et al. Psychiatric Presentation of Anti-NMDA Receptor Encephalitis. Front Neurol. 2019;10:1086.

66. Fauzi NAM, Joseph JP, Zain NRM, Hashim H. A severe anti-NMDA-receptor encephalitis case with extensive cortical and white matter changes, cerebral atrophy and communicating hydrocephalus. Proceedings of Singapore Healthcare. 2017;26(1):58-61.

67. Ferreira MG, Alcalde VL, Sánchez MHG, Hernández LH, Doyague Sánchez MJ. Successful treatment of anti-NMDA receptor encephalitis with early teratoma removal and plasmapheresis: A case report. Medicine (United States). 2018;97(31).

68. Fields J, Lim T, Kolevzon A, Coffey BJ. A rare case of anti-N-methyl-D-aspartate receptor encephalitis in an adolescent. J Child Adolesc Psychopharmacol. 2013;23(7):502-6.

69. Finné Lenoir X, Sindic C, van Pesch V, El Sankari S, de Tourtchaninoff M, Denays R, et al. Anti-N-methyl-D-aspartate receptor encephalitis with favorable outcome despite prolonged status epilepticus. Neurocrit Care. 2013;18(1):89-92.

70. Fisher J, Ellingson C, Tolby N. Psychosis in the ED: A case of NMDA receptor antibody encephalitis. Am J Emerg Med. 2017;35(7):1035.e5-.e6.

71. Flannery P, Yang I, Keyvani M, Sakoulas G. Acute Psychosis Due to Anti-N-Methyl D-Aspartate Receptor Encephalitis Following COVID-19 Vaccination: A Case Report. Front Neurol. 2021;12:764197.

72. Forrester A, Latorre S, O'Dea PK, Robinson C, Goldwaser EL, Trenton A, et al. Anti-NMDAR Encephalitis: A Multidisciplinary Approach to Identification of the Disorder and Management of Psychiatric Symptoms. Psychosomatics. 2020;61(5):456-66.

73. Frawley KJ, Calvo-Garcia MA, Krueger DA, McMasters RL. 'Benign' ovarian teratoma and N-methyl-D-aspartate receptor (NMDAR) encephalitis in a child. Pediatr Radiol. 2012;42(1):120-3.

74. Funayama M, Mizushima J, Takata T, Koreki A, Mimura M. Altered perception might be a symptom of anti-N-methyl-D-aspartate receptor (NMDAR) encephalitis. Neurocase. 2018;24(5-6):255-8.

75. Gao R, Zhang X, Bhekharee AK, Zhang Y. Solitary juxtacortical lesion associated with anti-N-methyl-D-aspartate receptor encephalitis: a case report. BMC Neurol. 2020;20(1):421.

76. Gastaldi M, Arbasino C, Dallocchio C, Diamanti L, Bini P, Marchioni E, et al. NMDAR encephalitis presenting as akinesia in a patient with Parkinson disease. J Neuroimmunol. 2019;328:35-7.

77. Gbadero DA, Adegbite EO, LePichon JB, Slusher TM. Case Presentation of Anti-NMDA Receptor Encephalitis in a 4-Year-Old Boy. J Trop Pediatr. 2018;64(4):352-4.

78. Goldberg EM, Taub KS, Kessler SK, Abend NS. Anti-NMDA receptor encephalitis presenting with focal non-convulsive status epilepticus in a child. Neuropediatrics. 2011;42(5):188-90.

79. Goldberg EM, Titulaer M, de Blank PM, Sievert A, Ryan N. Anti-N-methyl-D-aspartate receptor-mediated encephalitis in infants and toddlers: case report and review of the literature. Pediatr Neurol. 2014;50(2):181-4.

80. Goldberg TN, Cellucci MF. New Onset Insomnia in a Pediatric Patient: A Case of Anti-NMDA Receptor Encephalitis. Case Rep Pediatr. 2017;2017:4083785.

81. Gomes Ferreira M, Lapresa Alcalde V, García Sánchez MH, Hernández Hernández L, Doyague Sánchez MJ. Successful treatment of anti-NMDA receptor encephalitis with early teratoma removal and plasmapheresis: A case report. Medicine (Baltimore). 2018;97(31):e11325.

82. Govil-Dalela T, Datta I, Williams M. Refractory NMDA-receptor encephalitis in a teenager: A novel use of Bortezomib. J Neuroimmunol. 2021;355:577565.

83. Guo YH, Kuan TS, Hsieh PC, Lien WC, Chang CK, Lin YC. Rehabilitation for a child with recalcitrant anti-N-methyl-d-aspartate receptor encephalitis: case report and literature review. Neuropsychiatr Dis Treat. 2014;10:2263-7.

84. Hacohen Y, Wright S, Gadian J, Vincent A, Lim M, Wassmer E, et al. N-methyl-d-aspartate (NMDA) receptor antibodies encephalitis mimicking an autistic regression. Dev Med Child Neurol. 2016;58(10):1092-4.

85. Halbert RK. Anti-N-Methyl-D-Aspartate Receptor Encephalitis: A Case Study. J Neurosci Nurs. 2016;48(5):270-3.

86. Hallowell S, Tebedge E, Oates M, Hand E. Rituximab for Treatment of Refractory Anti-NMDA Receptor Encephalitis in a Pediatric Patient. J Pediatr Pharmacol Ther. 2017;22(2):118-23.

87. Han VX, Lee MWX, Lin JB. Multistage neurorehabilitation of an adolescent with severe anti-N-methyl-D-aspartate receptor encephalitis: a case report. Int J Rehabil Res. 2019;42(1):89-91.

88. Hao XS, Wang JT, Chen C, Hao YP, Liang JM, Liu SY. Effectiveness of Mycophenolate Mofetil in the Treatment of Pediatric Anti-NMDAR Encephalitis: A Retrospective Analysis of 6 Cases. Front Neurol. 2020;11:584446.

89. Hara M, Morita A, Kamei S, Yamaguchi M, Homma T, Nemoto N, et al. Anti-N-methyl-D-aspartate receptor encephalitis associated with carcinosarcoma with neuroendocrine differentiation of the uterus. J Neurol. 2011;258(7):1351-3.

90. Hashimoto R, Tagawa A, Komori N, Ogawa T, Kato H. Transient Dyschromatopsia, Static Form Agnosia, and Prosopagnosia Observed in a Patient with Anti-NMDA Receptor Encephalitis. Case Rep Neurol Med. 2019;2019:2929782.

91. Hatanaka M, Shimakawa S, Okumura A, Natsume J, Fukui M, Nomura S, et al. The efficacy of adrenocorticotropic hormone in a girl with anti-N-methyl-D-aspartate receptor encephalitis. Brain Dev. 2018;40(3):247-50.

92. Hattori Y, Yamashita Y, Mizuno M, Katano K, Sugiura-Ogasawara M, Matsukawa N. Anti-N-methyl-d-aspartate receptor limbic encephalitis associated with mature cystic teratoma of the fallopian tube. J Obstet Gynaecol Res. 2017;43(2):412-5.

93. Hau L, Csabi G, Rozsai B, Stankovics J, Tenyi T, Hollody K. Anti-N-methyl-D-aspartate receptor encephalitis and drug abuse - the probable role of molecular mimicry or the overstimulation of CB receptors in a 17-year-old adolescent - case report. Neuropsychopharmacol Hung. 2016;18(3):162-4.

94. Hébert J, El-Sadi F, Maurice C, Wennberg RA, Tang-Wai DF. Adult-Onset Anti-N-methyl-D-aspartate-receptor Encephalitis Presenting as a Non-Fluent Aphasia. Can J Neurol Sci. 2018;45(2):248-51.

95. Hegarty CP, Mikli JE. Behavioural disturbance requiring medical referral: A case of anti-N-methyl-D-aspartate receptor encephalitis in the emergency department. Emerg Med Australas. 2013;25(1):87-9.

96. Hegen H, Uprimny C, Grams A, Virgolini I, Ramberger M, Beer R, et al. Bi-insular cortical involvement in anti-NMDA-receptor encephalitis - a case report. BMC Neurol. 2016;16:130.

97. Hehar J, Otoupalova E, Grasso-Knight G. It's in the receptors: A rare cause of fever and psychosis. Chest. 2017;152(4):A361.

98. Heng KYC, Lee JH, Thomas T. Anti-N-Methyl-D-aspartate receptor encephalitis masquerading as fever of unknown origin. Clin Case Rep. 2021;9(4):2323-7.

99. Hermans T, Santens P, Matton C, Oostra K, Heylens G, Herremans S, et al. Anti-NMDA receptor encephalitis: still unknown and underdiagnosed by physicians and especially by psychiatrists? Acta Clin Belg. 2018;73(5):364-7.

100. Hinkle CD, Porter JN, Waldron EJ, Klein H, Tranel D, Heffelfinger A. Neuropsychological characterization of three adolescent females with anti-NMDA receptor encephalitis in the acute, post-acute, and chronic phases: an inter-institutional case series. Clin Neuropsychol. 2017;31(1):268-88.

101. Hofmann C, Baur MO, Schroten H. Anti-NMDA receptor encephalitis after TdaP-IPV booster vaccination: cause or coincidence? J Neurol. 2011;258(3):500-1.

102. Hole MK, Lennon VA, Cohen ML, Sokol DK. NMDA receptor encephalitis: Late treatment also effective. Pediatric Neurology. 2014;50(1):115-6.

103. Hopkins SA, Moodley KK, Chan D. Autoimmune limbic encephalitis presenting as relapsing psychosis. BMJ Case Rep. 2013;2013.

104. Hung TY, Foo NH, Lai MC. Anti-N-methyl-d-aspartate receptor encephalitis. Pediatr Neonatol. 2011;52(6):361-4.

105. Hur J. Fever of Unknown Origin: An Unusual Presentation of Anti-N-Methyl-D-Aspartate Receptor Encephalitis. Infect Chemother. 2015;47(2):129-32.

106. Iadisernia E, Battaglia FM, Vanadia E, Trapolino E, Vincent A, Biancheri R. Anti-N-methyl-D-aspartate-receptor encephalitis: cognitive profile in two children. Eur J Paediatr Neurol. 2012;16(1):79-82.

107. Ikeguchi R, Shibuya K, Akiyama S, Hino S, Kubo H, Takeda T, et al. Rituximab used successfully in the treatment of anti-NMDA receptor encephalitis. Intern Med. 2012;51(12):1585-9.

108. Ilyas F, Khalid M, Ilyas M, Khan Minhas AM, Qavi A. ANTI-NMDA RECEPTOR ENCEPHALITIS: A DIAGNOSTIC CHALLENGE FOR THE INTERNIST. Chest. 2020;158(4):A834.

109. Imai K, Fukuda T, Wada T, Kawanishi M, Yamauchi M, Hashiguchi Y, et al. Complete recovery from paraneoplastic anti-NMDAR encephalitis associated with a small ovarian teratoma following a laparoscopic salpingo-oophorectomy: A case report. Exp Ther Med. 2015;9(5):1723-6.

110. Imataka G, Yoshihara S. Immature ovarian teratoma with anti-NMDA-receptor encephalitis in a 13-year-old Japanese female patient. Med J Malaysia. 2021;76(3):436-7.

111. Irvine E, Cheng D, Xiao D. Atypical presentation of anti-N-methyl-D-aspartate receptor encephalitis in a 61-year-old Hispanic man. BMJ Case Rep. 2020;13(12).

112. Ishikawa Y, Ikeda K, Murata K, Hirayama T, Takazawa T, Yanagihashi M, et al. Ophthalmoplegia and flaccid paraplegia in a patient with anti-NMDA receptor encephalitis: a case report and literature review. Intern Med. 2013;52(24):2811-5.

113. Jan S, Anilkumar AC. Atypical Brain MRI Findings in a Child With Delayed Diagnosis of Anti-N-Methyl-D-Aspartate Receptor Encephalitis. Cureus. 2021;13(9):e18103.

114. Jandu AS, Odor PM, Vidgeon SD. Status epilepticus and anti-NMDA receptor encephalitis after resection of an ovarian teratoma. J Intensive Care Soc. 2016;17(4):346-52.

115. Jiang Y, Ma J, Gong T, Hao H, Chen H. The diffuse involvement of anti-N-methyl-D-aspartate receptor encephalitis in brain: a case report. BMC Neurol. 2019;19(1):230.

116. Jiménez-Zarazúa O, Martínez-Rivera MA, González-Carrillo PL, Vélez-Ramírez LN, Alcocer-León M, Tafoya-Rojas SXL, et al. Headache, Delirium or Encephalitis? A Case of Residual Mutism Secondary to Anti-NMDA Receptor Encephalitis. Case Rep Neurol. 2019;11(3):330-43.

117. Joe E, Desai J. An Atypical Case of Anti-N-Methyl-D-Aspartate Receptor Encephalitis. Pediatr Neurol. 2016;63:80-1.

118. Jones BP, Rees R, Saso S, Stalder C, Smith JR, Yazbek J. Ultrasound-guided laparoscopic ovarian preserving surgery to treat anti-NMDA receptor encephalitis. Bjog. 2017;124(2):337-41.

119. Jones KC, Schwartz AC, Hermida AP, Kahn DA. A Case of Anti-NMDA Receptor Encephalitis Treated with ECT. J Psychiatr Pract. 2015;21(5):374-80.

120. Jones SV, Breakey RW, Lockwood BM. A Case of Anti-NMDA Receptor Encephalitis With the Highest Reported CSF White Cells to Date. J Neuropsychiatry Clin Neurosci. 2014;26(4):E10-1.

121. Joseph K, Oliveira CR, Baltimore RS. A 14-Year-Old Girl with Slurred Speech, Aggressive Behavior, and Seizures. Pediatr Ann. 2015;44(6):236-7.

122. Justin Coffey M, Cooper JJ. Electroconvulsive Therapy in Anti-N-Methyl-D-Aspartate Receptor Encephalitis: A Case Report and Review of the Literature. Journal of ECT. 2016;32(4):225-9.

123. Kadoya M, Kadoya A, Onoue H, Ikewaki K, Kaida K. An Atypical Case of Anti-NMDA Receptor Encephalitis: Predominant Parkinsonism and Persisting Micrographia without Oro-facial Dyskinesia. Intern Med. 2015;54(15):1927-32.

124. Kadoya M, Onoue H, Kadoya A, Ikewaki K, Kaida K. Refractory status epilepticus caused by anti-NMDA receptor encephalitis that markedly improved following combination therapy with rituximab and cyclophosphamide. Intern Med. 2015;54(2):209-13.

125. Kaminski JA, Prüss H. N-methyl-d-aspartate receptor encephalitis with a reversible splenial lesion. European Journal of Neurology. 2019;26(6):e68-e9.

126. Kamran Mirza M, Pogoriler J, Paral K, Ananthanarayanan V, Mandal S, Mazin A, et al. Adjunct therapeutic plasma exchange for anti-N-methyl- D -aspartate receptor antibody encephalitis: A case report and review of literature. Journal of Clinical Apheresis. 2011;26(6):362-5.

127. Kasahara H, Sato M, Nagamine S, Makioka K, Tanaka K, Ikeda Y. Temporal Changes on (123)I-Iomazenil and Cerebral Blood Flow Single-photon Emission Computed Tomography in a Patient with Anti-N-methyl-D-aspartate Receptor Encephalitis. Intern Med. 2019;58(10):1501-5.

128. Kashihara T, Nozaki I, Sakai K, Minamikawa J, Nakamura-Shindo K, Akagi A, et al. Recovery from multidisciplinary therapy-refractory anti-NMDA receptor encephalitis after over three years of mechanical ventilation. Clin Neurol Neurosurg. 2021;202:106477.

129. Kashyape P, Taylor E, Ng J, Krishnakumar D, Kirkham F, Whitney A. Successful treatment of two paediatric cases of anti-NMDA receptor encephalitis with cyclophosphamide: the need for early aggressive immunotherapy in tumour negative paediatric patients. Eur J Paediatr Neurol. 2012;16(1):74-8.

130. Kataoka H, Sawa N, Tonomura Y, Ueno S. Early progression of brain atrophy in patients with anti-N-methyl-D - Aspartate receptor encephalitis. Medicine (United States). 2017;96(17).

131. Kataoka H, Takatani T, Ueno S. Low-voltage EEG activity presenting from psychotic stage in a patient with anti-NMDA receptor encephalitis. BMJ Case Rep. 2012;2012.

132. Kattepur AK, Patil D, Shankarappa A, Swamy S, Chandrashekar NS, Chandrashekar P, et al. Anti-NMDAR limbic encephalitis - A clinical curiosity. World Journal of Surgical Oncology. 2014;12(1).

133. Kaur H, Mittal GK. Anti-N-methyl-D-aspartate receptor encephalitis unresponsive to early and aggressive immunotherapy in a young female: A case report. Clinical and Experimental Neuroimmunology. 2020;11(4):233-7.

134. Kayal AK, Das M, Bhowmick S, Synmon B. Relapsing Anti-NMDAR Encephalitis after a gap of eight years in a girl from North-East India. Ann Indian Acad Neurol. 2014;17(3):349-51.

135. Keddie S, Crisp SJ, Blackaby J, Cox A, Coles A, Hart M, et al. Plasma cell depletion with bortezomib in the treatment of refractory N-methyl-d-aspartate (NMDA) receptor antibody encephalitis. Rational developments in neuroimmunological treatment. Eur J Neurol. 2018;25(11):1384-8.

136. Keller S, Roitman P, Ben-Hur T, Bonne O, Lotan A. Anti-NMDA Receptor Encephalitis Presenting as an Acute Psychotic Episode in a Young Woman: An Underdiagnosed yet Treatable Disorder. Case Rep Psychiatry. 2014;2014:868325.

137. Kelly LJ, Sexton VL. Anti-NMDAR Encephalitis. Neurodiagn J. 2016;56(2):101-8.

138. Khoo CS, Zulkifli NH, Rahman SSA. An unusual case of refractory status epilepticus in a young lady: anti-NMDA receptor encephalitis. Clin Med (Lond). 2017;17(5):436-8.

139. Khoodoruth MAS, Chut-Kai Khoodoruth WN, Alshawwaf MKM. Anti-N-methyl-D-aspartate receptor encephalitis in a young female with subclinical hypothyroidism associated with anti-thyroid peroxidase and anti-thyroglobulin antibodies: A case report. SAGE Open Med Case Rep. 2020;8:2050313x20949780.

140. Khundakji Y, Masri A, Khuri-Bulos N. Anti-NMDA receptor encephalitis in a toddler: A diagnostic challenge. Int J Pediatr Adolesc Med. 2018;5(2):75-7.

141. Kim E, Park EG, Lee J, Lee M, Kim J, Lee J. Anti-NMDAR Encephalitis in a 13-Year-Old Female: A 24-Month Clinical Follow-Up. J Epilepsy Res. 2018;8(1):41-8.

142. Kim EH, Kim YJ, Ko TS, Yum MS, Lee JH. A young child of anti-NMDA receptor encephalitis presenting with epilepsia partialis continua: the first pediatric case in Korea. Korean J Pediatr. 2016;59(Suppl 1):S133-s8.

143. Kim H, Ryu H, Kang JK. Anti-NMDA Receptor Antibody Encephalitis Presenting with Unilateral Non-convulsive Status Epilepticus in a Male Patient. J Epilepsy Res. 2015;5(1):17-9.

144. Kim LN, Edwards L, Goonetilleke N, Kane A, McDougall A, Calic Z. Bortezomib for the treatment of refractory anti-N-methyl-d-aspartate receptor encephalitis. Intern Med J. 2020;50(12):1591-2.

145. Kirkpatrick MP, Clarke CD, Sonmezturk HH, Abou-Khalil B. Rhythmic delta activity represents a form of nonconvulsive status epilepticus in anti-NMDA receptor antibody encephalitis. Epilepsy Behav. 2011;20(2):392-4.

146. Koolwal A, Agrawal S, Koolwal G, Saxena K, Daral A. Anti-N-methyl-D-aspartate receptor encephalitis: Case series of psychiatric presentations. Annals of Indian Academy of Neurology. 2020;23(2):225-7.

147. Kumar S, Fan Z, Greenwood RS. Anti-NMDA receptor encephalitis subacute cerebellitis remarkably responsive to rituximab. Journal of Pediatric Neurology. 2013;11(1):35-8.

148. Kung DH, Qiu C, Kass JS. Psychiatric manifestations of anti-NMDA receptor encephalitis in a man without tumor. Psychosomatics. 2011;52(1):82-5.

149. Kuo YL, Tsai HF, Lai MC, Lin CH, Yang YK. Anti-NMDA receptor encephalitis with the initial presentation of psychotic mania. J Clin Neurosci. 2012;19(6):896-8.

150. Kuppuswamy PS, Takala CR, Sola CL. Management of psychiatric symptoms in anti-NMDAR encephalitis: a case series, literature review and future directions. Gen Hosp Psychiatry. 2014;36(4):388-91.

151. Kurian M, Fluss J, Korff C. Anti-NMDA receptor encephalitis: the importance of early diagnosis and aggressive immunotherapy in tumor negative pediatric patients. Eur J Paediatr Neurol. 2012;16(6):764-5.

152. Kwon HS, Jo SH, Park Y, Choi H, Lee YJ, Koh SH, et al. A Case of Anti-NMDA Receptor Encephalitis with Normal Findings on Initial Diagnostic Tests. Dement Neurocogn Disord. 2020;19(1):28-30.

153. Labate A, Quattrone A, Dalmau J, Gambardella A. Anti-N-methyl-D-aspartate-glutamic-receptor encephalitis presenting as paroxysmal exercise-induced foot weakness. Mov Disord. 2013;28(6):820-2.

154. Lapébie FX, Kennel C, Magy L, Projetti F, Honnorat J, Pichon N, et al. Potential side effect of propofol and sevoflurane for anesthesia of anti-NMDA-R encephalitis. BMC Anesthesiol. 2014;14:5.

155. Lasoff DR, Corbett-Detig J, Sell R, Nolan M, Wardi G. Anti-N-Methyl-D-Aspartate Receptor Encephalitis, an Underappreciated Disease in the Emergency Department. West J Emerg Med. 2016;17(3):280-2.

156. Lazzarin SM, Vabanesi M, Cecchetti G, Fazio R, Fanelli GF, Volonté MA, et al. Refractory anti-NMDAR encephalitis successfully treated with bortezomib and associated movements disorders controlled with tramadol: a case report with literature review. J Neurol. 2020;267(8):2462-8.

157. Lebon S, Mayor-Dubois C, Popea I, Poloni C, Selvadoray N, Gumy A, et al. Anti-N-methyl-D-aspartate (NMDA) receptor encephalitis mimicking a primary psychiatric disorder in an adolescent. J Child Neurol. 2012;27(12):1607-10.

158. Leding C, Marstrand L, Jorgensen A. Complete cognitive recovery in a severe case of anti-N-methyl-d-aspartate receptor encephalitis treated with electroconvulsive therapy. BMJ Case Rep. 2020;13(2).

159. Lee CH, Kim EJ, Lee MH, Yim GW, Kim KJ, Kim KK, et al. Anti-N-methyl-D-aspartate Receptor Encephalitis: a Rare Complication of Ovarian Teratoma. J Korean Med Sci. 2020;35(24):e207.

160. Lee J, Kang S, Chang HJ, Lee YH, Son JH, Kong TW, et al. Anti-N-methyl-D-aspartate receptor encephalitis associated with ovarian teratoma in Korea: three case reports. Yeungnam Univ J Med. 2021;38(4):350-5.

161. Lee KW, Liou LM, Wu MN. Fulminant course in a patient with anti-N-methyl-D-aspartate receptor encephalitis with bilateral ovarian teratomas: A case report and literature review. Medicine (Baltimore). 2018;97(15):e0339.

162. Lee LH, Lu CJ. Long-term and Strong Immunotherapy to Treat Anti-N-Methyl- D-Aspartate Receptor Encephalitis with Refractory Status Epilepticus. Acta Neurol Taiwan. 2016;25(3):99-103.

163. Leel N, Thakkar HS, Drake D, Bouhadiba N. Ovarian teratoma associated with anti-NMDA (N-methyl D-aspartate) receptor encephalitis. BMJ Case Rep. 2018;2018.

164. Li C, Liu C, Lin F, Liu L. Anti-N-methyl-D-aspartate receptor encephalitis associated with mediastinal teratoma: a rare case report and literature review. J Thorac Dis. 2017;9(12):E1118-e21.

165. Li C, Meng F, Gong S. Clinical analysis of five case reports of ovarian teratoma withanti-N-methyl-D-aspartate receptor encephalitis. Ann Palliat Med. 2021;10(4):4950-4.

166. Li H, Guo YK, Cui YL, Peng T. Anti-N-methyl-D-aspartate receptor encephalitis: A case report. Medicine (Baltimore). 2018;97(50):e13625.

167. Li R, Jiang L, Li XJ, Hong SQ, Zhong M, Hu Y. Analysis and discussion of the rare complication of autoimmune encephalitis: Two case reports. Medicine (Baltimore). 2018;97(27):e11202.

168. Li S, Zhao A. A case of anti-NMDAR encephalitis induced by ovarian teratoma. Cell Biochem Biophys. 2015;71(2):1011-4.

169. Li W, Jia D, Tong L, Lun Z, Li H. Anti-N-methyl-D-aspartate receptor encephalitis induced by bilateral ovarian teratomas with distinct histopathologic types: A case report and brief literature review. Medicine (Baltimore). 2019;98(48):e18148.

170. Li Y, Wang Q, Liu C, Wu Y. Anti-N-Methyl-d-Aspartate Receptor Encephalitis in a Patient with Alcoholism: A Rare Case Report. Front Psychiatry. 2017;8:141.

171. Liang Z, Yang S, Sun X, Li B, Li W, Liu Z, et al. Teratoma-associated anti-NMDAR encephalitis: Two cases report and literature review. Medicine (Baltimore). 2017;96(49):e9177.

172. Lineberry O, Patil A, Bailley K. A case report: 38-year-old female with anti-NMDA-receptor encephalitis without ovarian teratoma or occult malignancy. Chest. 2013;144(4).

173. Liu H, Jian M, Liang F, Yue H, Han R. Anti-N-methyl-D-aspartate receptor encephalitis associated with an ovarian teratoma: two cases report and anesthesia considerations. BMC Anesthesiol. 2015;15:150.

174. Liu LY, Wang YY, Pang LY, Zhang GX, Zou LP. Anti-N-Methyl-D-Aspartate Receptor Encephalitis in a 3-Year-Old Toddler with the Involvement of Severe Autonomic Dysfunctions in Gastrointestinal System: A Case Report. Journal of Pediatric Neurology. 2019;17(1):41-4.

175. Low JM. Important differential in a patient presenting with neuropsychiatric symptoms: Anti-N-Methyl-D-Aspartate receptor encephalitis. Med J Malaysia. 2017;72(5):306-7.

176. Lu JP, Song XK, Li HY, Wang GP. Anti-N-methyl-D-aspartate receptor encephalitis in a 17-year-old female patient with 3 years of follow-up. Chin Med J (Engl). 2019;132(8):996-7.

177. Luca N, Daengsuwan T, Dalmau J, Jones K, deVeber G, Kobayashi J, et al. Anti-N-methyl-D-aspartate receptor encephalitis: a newly recognized inflammatory brain disease in children. Arthritis Rheum. 2011;63(8):2516-22.

178. Lwanga A, Kamson DO, Wilkins TE, Sharma V, Schulte JJ, Miller J, et al. Occult teratoma in a case of N-methyl-D-aspartate receptor encephalitis. Neuroradiol J. 2018;31(4):415-9.

179. Lwin S, San Yi M, Mardiana K, Woon SY, Nwe TM. Ovarian teratoma-associated anti-NMDAR encephalitis in a 12-year-old girl. Med J Malaysia. 2020;75(6):731-3.

180. Maccaferri GE, Rossetti AO, Dalmau J, Berney A. Anti-N-Methyl-D-Aspartate Receptor Encephalitis: A New Challenging Entity for Consultation-Liaison Psychiatrist. Brain Disord Ther. 2016;5(2).

181. Maggina P, Mavrikou M, Karagianni S, Skevaki CL, Triantafyllidou A, Voudris C, et al. Anti-N-methyl-D-aspartate receptor encephalitis presenting with acute psychosis in a preteenage girl: a case report. J Med Case Rep. 2012;6:224.

182. Maggio MC, Mastrangelo G, Skabar A, Ventura A, Carrozzi M, Santangelo G, et al. Atypical presentation of anti-N-methyl-D-aspartate receptor encephalitis: two case reports. J Med Case Rep. 2017;11(1):225.

183. Mahale R, Farsana MK, Mahadevan A, Mukherjee J, Lakshmi V, Sandeep M, et al. Stroke-like episodes with cerebellar ataxia as presenting manifestation of adult-onset anti-N-methyl D-aspartate receptor encephalitis: an unusual presentation. Acta Neurol Belg. 2021;121(4):1093-5.

184. Malayev Y, Alberts J, Verardi MA, Mattison AR, Imlay S. Immature Teratoma Associated With Anti-N-Methyl-D-Aspartate Receptor Encephalitis. J Am Osteopath Assoc. 2015;115(9):573-7.

185. Mann A, Machado NM, Liu N, Mazin AH, Silver K, Afzal KI. A multidisciplinary approach to the treatment of anti-NMDA-receptor antibody encephalitis: a case and review of the literature. J Neuropsychiatry Clin Neurosci. 2012;24(2):247-54.

186. Maqbool M, Oleske DA, Huq AH, Salman BA, Khodabakhsh K, Chugani HT. Novel FDG-PET findings in anti-NMDA receptor encephalitis: a case based report. J Child Neurol. 2011;26(10):1325-8.

187. Marinova Z, Bausch-Becker N, Savaskan E. Anti-N-methyl-d-aspartate receptor encephalitis in an older patient presenting with a rapid onset of delusions and amnesia. BMJ Case Rep. 2019;12(4).

188. Marques IB, Teotónio R, Cunha C, Bento C, Sales F. Anti-NMDA receptor encephalitis presenting with total insomnia - A case report. Journal of the Neurological Sciences. 2014;336(1-2):276-80.

189. Masghati S, Nosratian M, Dorigo O. Anti-N-methyl-aspartate receptor encephalitis in identical twin sisters: role for oophorectomy. Obstet Gynecol. 2014;123(2 Pt 2 Suppl 2):433-5.

190. Matoq AA, Rappoport AS, Yang Y, O'Babatunde J, Bakerywala R, Sheth RD. Anti-NMDA-receptor antibody encephalitis in infants. Epilepsy Behav Case Rep. 2015;4:99-101.

191. Matsumoto H, Hashida H, Takahashi Y. Dystonic Seizures and Intense Hyperperfusion of the Basal Ganglia in a Patient with Anti-N-Methyl-D-Aspartate Receptor Encephalitis. Case Rep Neurol. 2017;9(3):272-6.

192. Mechelhoff D, van Noort BM, Weschke B, Bachmann CJ, Wagner C, Pfeiffer E, et al. Anti-NMDA receptor encephalitis presenting as atypical anorexia nervosa: an adolescent case report. Eur Child Adolesc Psychiatry. 2015;24(11):1321-4.

193. Medepalli K, Lee CM, Benninger LA, Elwing JM. Psychosis: call a surgeon? A rare etiology of psychosis requiring resection. SAGE Open Med Case Rep. 2016;4:2050313x16670084.

194. Meixensberger S, Tebartz van Elst L, Schweizer T, Maier SJ, Prüss H, Feige B, et al. Anti-N-Methyl-D-Aspartate-Receptor Encephalitis: A 10-Year Follow-Up. Front Psychiatry. 2020;11:245.

195. Menon D, Menon RN, Kesavadas C, Mahadevan A, Radhakrishnan A, Kannoth S, et al. Clinical-radiological-pathological correlation in an unusual case of refractory epilepsy: a two-year journey of whodunit! Epileptic Disord. 2018;20(1):51-9.

196. Mitra AD, Afify A. Ovarian teratoma associated Anti-N-methyl-D-aspartate receptor encephalitis: a difficult diagnosis with a favorable prognosis. Autops Case Rep. 2018;8(2):e2018019.

197. Miyauchi A, Monden Y, Osaka H, Takahashi Y, Yamagata T. A case of anti-NMDAR encephalitis presented hypotensive shock during plasma exchange. Brain Dev. 2016;38(4):427-30.

198. Moldavski A, Wenz H, Lange BE, Rohleder C, Leweke FM. Case Report: Severe Adolescent Major Depressive Syndrome Turns Out to Be an Unusual Case of Anti-NMDA Receptor Encephalitis. Front Psychiatry. 2021;12:679996.

199. Morales-Briceño H, Fung VSC. Isolated Nocturnal Occurrence of Orofacial Dyskinesias in N-methyl-D-aspartate Receptor Encephalitis-A New Diagnostic Clue. Mov Disord Clin Pract. 2017;4(6):884-6.

200. Mori L, Campanella W, Vestito L, Marinelli L, Benedetti L, Cocito L, et al. I can't count, but I can beat you playing cards: a case report on autoimmune encephalitis. BMC Neurol. 2021;21(1):347.

201. Moss N, Petranovich CL, Parks L, Sherwood A. Two Case Reports of Neuropsychological Outcomes following Pediatric anti-N-methyl D-aspartate Receptor Autoimmune Encephalitis. Dev Neuropsychol. 2018;43(7):656-68.

202. Motohara T, Tayama S, Narantuya D, Tashiro H, Katabuchi H. Anti-N-methyl-d-aspartate receptor encephalitis associated with ovarian teratoma: Clinical presentation, diagnosis, treatment, and surgical management. International Cancer Conference Journal. 2013;2(3):121-30.

203. Moura M, Silva-Dos-Santos A, Afonso J, Talina M. First-episode psychosis in a 15 year-old female with clinical presentation of anti-NMDA receptor encephalitis: a case report and review of the literature. BMC Res Notes. 2016;9:374.

204. Moussa T, Afzal K, Cooper J, Rosenberger R, Gerstle K, Wagner-Weiner L. Pediatric anti-NMDA receptor encephalitis with catatonia: treatment with electroconvulsive therapy. Pediatr Rheumatol Online J. 2019;17(1):8.

205. Mugavin M, Mueller BH, Desai M, Golnik KC. Optic Neuropathy As the Initial Presenting Sign of N-methyl-d-aspartate (NMDA) Encephalitis. Neuro-Ophthalmology. 2017;41(2):90-3.

206. Mutti C, Barocco F, Zinno L, Negrotti A, Spallazzi M, Pavesi G, et al. A case of reversible anti-NMDA-receptor encephalitis: neuropsychological and neuroradiological features. Neurol Sci. 2017;38(12):2231-6.

207. Naik A, Prakash S, Ray GK, Mukherjee S. Variable response to therapeutic plasma exchange in pediatric anti-NMDA receptor encephalitis. Transfus Clin Biol. 2021;28(3):287-90.

208. Naoura I, Didelot A, Walker F, Luton D, Koskas M. Anti-N-methyl-D-aspartate receptor encephalitis complicating ovarian teratomas: a case report. Am J Obstet Gynecol. 2011;205(4):e6-8.

209. Nazari R, Carmona CA. Rapid onset of altered mental status with progressive autonomic instability and hypoventilation in a 2-year-old male. Turkish Journal of Pediatrics. 2019;61(6):949-52.

210. Neerukonda N, Bliss M, Jafroodifar A, Leontieva L. Olanzapine and Lorazepam Used in the Symptomatic Management of Excited Catatonia Secondary to Anti-N-Methyl-D-Aspartate Receptor Encephalitis. Cureus. 2020;12(6):e8689.

211. Ng AC, Tripic M, Mirsattari SM. Teratoma-negative anti-NMDA receptor encephalitis presenting with a single generalized tonic-clonic seizure. Epilepsy Behav Case Rep. 2018;10:29-31.

212. Nijmeijer S, Bontemps S, Naeije L, Coutinho J. Anti-N-methyl-D-aspartate receptor encephalitis in a pre-teenage girl: a case report. Eur J Pediatr. 2014;173(5):681-3.

213. Nillo RM, Broce IJ, Uzgil B, Singhal NS, Glastonbury CM, Hess CP, et al. Longitudinal analysis of regional brain changes in anti-NMDAR encephalitis: a case report. BMC Neurol. 2021;21(1):412.

214. Nizam A, Menzin AW, Whyte JS. Anti-NMDA receptor encephalitis with neurologic sequelae refractory to conservative therapy with complete response to adjuvant therapy. Gynecol Oncol Rep. 2020;33:100597.

215. Norton D, Pesce MS, Gill D. A Pounding Problem: A Case of Recurrent Headache Caused by Anti-NMDA Receptor Encephalitis. J Emerg Med. 2021;60(3):345-8.

216. Obara K, Ono T, Toyoshima I. Anti-N-Methyl-D-Aspartate Receptor Encephalitis with Decrease in Blood Flow in Cerebellum. Case Rep Neurol. 2021;13(1):17-23.

217. Obi CA, Thompson E, Mordukhaev L, Khan I, Zhang NJ. Anti-N-methyl-d-aspartate receptor encephalitis triggered by emotional stress. Proc (Bayl Univ Med Cent). 2019;32(4):605-6.

218. Olaleye KT, Oladunjoye AO, Otuada D, Anugwom GO, Basiru TO, Udeogu JE, et al. The Effectiveness of Electroconvulsive Therapy on Catatonia in a Case of Anti-N-Methyl-D-Aspartate (Anti-NMDA) Receptor Encephalitis. Cureus. 2021;13(6):e15706.

219. Omata T, Kodama K, Watanabe Y, Iida Y, Furusawa Y, Takashima A, et al. Ovarian teratoma development after anti-NMDA receptor encephalitis treatment. Brain Dev. 2017;39(5):448-51.

220. Omura T, Sonoda S, Nagata K, Okita T, Hoshiai A, Sano H, et al. Anti-NMDAR encephalitis: case report and diagnostic issues. Acute Med Surg. 2015;2(1):56-9.

221. Onpoaree N, Veeravigrom M, Sanpavat A, Suratannon N, Sintusek P. Unremitting diarrhoea in a girl diagnosed anti-N-methyl-D-aspartate-receptor encephalitis: A case report. World J Clin Cases. 2020;8(20):4866-75.

222. Palakkuzhiyil N, Uvais NA, Moideen S, Shihabudheen P. Anti-NMDA-receptor encephalitis presenting with catatonia in a middle aged male. Asian J Psychiatr. 2018;35:26-7.

223. Pattanayak P, Solnes LB. Paraneoplastic Syndrome With Anti-NMDAR Encephalitis Associated With Ovarian Teratomas. Clin Nucl Med. 2017;42(2):e128-e9.

224. Pavone P, Falsaperla R, Ruggieri M, Verrotti A, Lubrano R, Rizzo R, et al. Clinical Course of N-Methyl-D-Aspartate Receptor Encephalitis and the Effectiveness of Cyclophosphamide Treatment. Journal of Pediatric Neurology. 2017;15(2):84-9.

225. Pham HP, Daniel-Johnson JA, Stotler BA, Stephens H, Schwartz J. Therapeutic plasma exchange for the treatment of anti-NMDA receptor encephalitis. J Clin Apher. 2011;26(6):320-5.

226. Player B, Harmelink M, Bordini B, Weisgerber M, Girolami M, Croix M. Pediatric Opsoclonus-Myoclonus-Ataxia Syndrome Associated With Anti-N-methyl-D-aspartate Receptor Encephalitis. Pediatr Neurol. 2015;53(5):456-8.

227. Ponte A, Brito A, Nóbrega C, Pinheiro S, Gama Marques J. Catatonia in anti-N-methyl-D-aspartate (NMDA) receptor encephalitis misdiagnosed as schizophrenia. Acta Medica Portuguesa. 2019;32(13).

228. Poorthuis MHF, van Rooij JLM, Koch AH, Verdonkschot AEM, Leembruggen MM, Titulaer MJ. Cerebellar ataxia as a presenting symptom in a patient with anti-NMDA receptor encephalitis. Neurol Neuroimmunol Neuroinflamm. 2019;6(4):e579.

229. Power L, James J, Masoud I, Altman A. Tubal teratoma causing anti-NMDAR encephalitis. J Obstet Gynaecol Can. 2014;36(12):1093-6.

230. Prithvi A, Kesavan S, Saini AG, Suthar R, Mahadevan A. Little Children, Bigger Problems: Anti-N-Methyl D-Aspartate Receptor Encephalitis! Indian J Pediatr. 2019;86(11):1048-50.

231. Punja M, Pomerleau AC, Devlin JJ, Morgan BW, Schier JG, Schwartz MD. Anti-N-methyl-D-aspartate receptor (anti-NMDAR) encephalitis: an etiology worth considering in the differential diagnosis of delirium. Clin Toxicol (Phila). 2013;51(8):794-7.

232. Raha S, Gadgil P, Sankhla C, Udani V. Nonparaneoplastic anti-N-methyl-D-aspartate receptor encephalitis: a case series of four children. Pediatr Neurol. 2012;46(4):246-9.

233. Rainey K, Gholkar B, Cheesman M. Anti-NMDA receptor encephalitis: an easily missed diagnosis in older patients. Age Ageing. 2014;43(5):725-6.

234. Rakiro J, Sokhi D. Fatal Autoimmune Anti-NMDA-Receptor Encephalitis with Poor Prognostication Score in a Young Kenyan Female. Int Med Case Rep J. 2021;14:343-7.

235. Ratuszny D, Skripuletz T, Wegner F, Groß M, Falk C, Jacobs R, et al. Case Report: Daratumumab in a Patient With Severe Refractory Anti-NMDA Receptor Encephalitis. Front Neurol. 2020;11:602102.

236. Reji R, Alexander S, Vijayan M. A case series on anti nmdar encephalitis. International Journal of Pharmacy and Pharmaceutical Sciences. 2017;9(4):220-2.

237. Ren C, Nai Y, Lv W, Liu H, Chen Q, Sun ZW, et al. Focus on autonomic dysfunctions in anti-NMDAR encephalitis: a case report. Eur Rev Med Pharmacol Sci. 2019;23(24):10970-5.

238. Restrepo-Martínez M, Chacón-González J, Bayliss L, Ramírez-Bermúdez J, Fricchione GL, Espinola-Nadurille M. Delirious Mania as a Neuropsychiatric Presentation in Patients With Anti-N-methyl-D-aspartate Receptor Encephalitis. Psychosomatics. 2020;61(1):64-9.

239. Rita CG, Nieto Gañan I, Jimenez Escrig A, Carrasco Sayalero Á. Anti-N-Methyl-D-Aspartate Encephalitis as Paraneoplastic Manifestation of Germ-Cells Tumours: A Cases Report and Literature Review. Case Reports Immunol. 2019;2019:4762937.

240. Roberts C, McEachern M, Mounsey A. CSF studies which ultimately led to the possible diagnosis of anti-NMDAR encephalitis. BMJ Case Rep. 2020;13(5).

241. Roberts R, MacDougall NJ, O'Brien P, Abdelaziz K, Christie J, Swingler R. Not hysteria: ovarian teratoma-associated anti-N-methyl-D-aspartate receptor encephalitis. Scott Med J. 2012;57(3):182.

242. Rollas T, Yıldız Mİ, Özçelik Eroğlu E, Aytulun A, Arslan GA, Tuncer MA, et al. P.371 Rituximab as a Third Generation Antipsychotic Agent: A Clinical Case. European Neuropsychopharmacology. 2020;40:S213-S4.

243. Rong X, Xiong Z, Cao B, Chen J, Li M, Li Z. Case report of anti-N-methyl-D-aspartate receptor encephalitis in a middle-aged woman with a long history of major depressive disorder. BMC Psychiatry. 2017;17(1):320.

244. Rozier M, Morita D, King M. Anti-N-Methyl-D-Aspartate Receptor Encephalitis: A Potential Mimic of Neuroleptic Malignant Syndrome. Pediatr Neurol. 2016;63:71-2.

245. Rutledge S, Chalissery A, O'Connor R, Mahon S, Connolly S, Farrell M, et al. Anti-NMDA-receptor antibody-mediated cortical blindness: a case report. Qjm. 2016;109(2):127-8.

246. Ryan SA, Costello DJ, Cassidy EM, Brown G, Harrington HJ, Markx S. Anti-NMDA receptor encephalitis: a cause of acute psychosis and catatonia. J Psychiatr Pract. 2013;19(2):157-61.

247. Sabbula BR, Yemmanur S, Sanivarapu R, Kagolanu D, Shadab A. Finding the Cause of Psychosis: A Challenging Case of Anti-NMDAR Encephalitis. Case Rep Med. 2020;2020:2074704.

248. Safadieh L, Dabbagh O. Anti-N-methyl-D-aspartate (NMDA) receptor encephalitis in a young Lebanese girl. J Child Neurol. 2013;28(10):1222-5.

249. Sakamoto H, Hirano M, Samukawa M, Ueno S, Maekura S, Fujimura H, et al. Details of treatment-related difficulties in men with anti-N-methyl-D-aspartate receptor encephalitis. Eur Neurol. 2013;69(1):21-6.

250. Salehi N, Yuan AK, Stevens G, Koshy R, Klein WF. A Case of Severe Anti-N-Methyl D-Aspartate (Anti-NMDA) Receptor Encephalitis with Refractory Autonomic Instability and Elevated Intracranial Pressure. Am J Case Rep. 2018;19:1216-21.

251. Salvucci A, Devine IM, Hammond D, Sheth RD. Pediatric anti-NMDA (N-methyl D-aspartate) receptor encephalitis. Pediatr Neurol. 2014;50(5):507-10.

252. Sameshima A, Hidaka T, Shima T, Nakashima A, Hasegawa T, Saito S. Anti-N-methyl-D-aspartate receptor encephalitis associated with ovarian immature teratoma. J Obstet Gynaecol Res. 2011;37(12):1883-6.

253. Sancho-Saúco J, Corraliza-Galán V, Lázaro-Carrasco de la Fuente J, Sánchez-Martínez C, Pelayo-Delgado I, De Pablos-Antona MJ, et al. Anti-NMDA receptor encephalitis: two case reports associated with ovarian teratoma and a literature review. J Obstet Gynaecol. 2019;39(6):864-5.

254. Sanmaneechai O, Song JL, Nevadunsky N, Moshé SL, Overby PJ. Anti-N-methyl-d-aspartate encephalitis with ovarian cystadenofibroma. Pediatr Neurol. 2013;48(3):232-5.

255. Schiavi MC, Manganelli F, Morgani C, Cignini P, Yacoub V, Carletti V, et al. Mature Ovarian Teratoma: Neurological Implications in a Young Woman. Case Rep Obstet Gynecol. 2021;2021:3085559.

256. Schulz DC, Pandey SK, Bursztyn L. Optic Nerve Atrophy in N-methyl-D-aspartate (NMDA) Encephalitis. Can J Neurol Sci. 2020;47(1):139-41.

257. Scott O, Richer L, Forbes K, Sonnenberg L, Currie A, Eliyashevska M, et al. Anti-N-methyl-D-aspartate (NMDA) receptor encephalitis: an unusual cause of autistic regression in a toddler. J Child Neurol. 2014;29(5):691-4.

258. See AT, Woo YL, Crawford R. Acute encephalitis secondary to an ovarian teratoma. J Obstet Gynaecol. 2012;32(6):604-6.

259. Seward S. Anti-N-methyl-D-aspartate receptor antibody encephalitis: An important cause of encephalitis in young adults. A report of two cases. J Am Coll Health. 2019;67(1):1-3.

260. Sharma B, Handa R, Prakash S, Nagpal K, Gupta P. Anti-NMDA receptor encephalitis: a neurological disease in psychiatric disguise. Asian J Psychiatr. 2014;7(1):92-4.

261. Sharma P, Sagar R, Patra B, Saini L, Gulati S, Chakrabarty B. Psychotic symptoms in anti-N-methyl-d-aspartate (NMDA) receptor encephalitis: A case report and challenges. Asian J Psychiatr. 2016;22:135-7.

262. Shimoyama Y, Umegaki O, Agui T, Kadono N, Minami T. Anti-NMDA receptor encephalitis presenting as an acute psychotic episode misdiagnosed as dissociative disorder: a case report. JA Clin Rep. 2016;2(1):22.

263. Simabukuro MM, Freitas CHA, Castro LHM. A patient with a long history of relapsing psychosis and mania presenting with anti-NMDA receptor encephalitis ten years after first episode. Dement Neuropsychol. 2015;9(3):311-4.

264. Simabukuro MM, Watanabe RGS, Pinto LF, Guariglia C, Gonçalves D, Anghinah R. A successful case of anti-NMDAR encephalitis without tumor treated with a prolonged regimen of plasmapheresis. Dement Neuropsychol. 2014;8(1):87-9.

265. Simmons ML, Perez KA. Bortezomib for treatment of anti-NMDA receptor encephalitis in a pediatric patient refractory to conventional therapy. Am J Health Syst Pharm. 2021;78(5):395-400.

266. Soares EMV, Kauark RBG, Rocha MSG, Brucki SMD. Anti-NMDA-R encephalitis: follow-up of 24 months. Dement Neuropsychol. 2013;7(3):304-7.

267. Sommeling C, Santens P. Anti-N-methyl-D-aspartate (anti-NMDA) receptor antibody encephalitis in a male adolescent with a large mediastinal teratoma. J Child Neurol. 2014;29(5):688-90.

268. Subramaniam S. A confused child. Clinical Pediatric Emergency Medicine. 2015;16(1):69-74.

269. Suthar R, Sankhyan N, Singhi P. Hyperkinetic Movement Disorder in a Girl with Anti-NMDA Receptor Encephalitis. Indian Pediatr. 2016;53(1):81.

270. Sveinsson O, Granqvist M, Forslin Y, Blennow K, Zetterberg H, Piehl F. Successful combined targeting of B- and plasma cells in treatment refractory anti-NMDAR encephalitis. J Neuroimmunol. 2017;312:15-8.

271. Tamma PD, Agwu AL, Hartman AL. Behavior outbursts, orofacial dyskinesias, and CSF pleocytosis in a healthy child. Pediatrics. 2011;128(1):e242-5.

272. Tanyi JL, Marsh EB, Dalmau J, Chu CS. Reversible paraneoplastic encephalitis in three patients with ovarian neoplasms. Acta Obstet Gynecol Scand. 2012;91(5):630-4.

273. Tavasoli AR, Shahidi G, Parvaresh M, Fasano A, Ashrafi MR, Hosseinpour S, et al. Deep brain stimulation in status dystonicus caused by anti-NMDA receptor encephalitis. Parkinsonism Relat Disord. 2019;66:255-7.

274. Teixeira S, Caldeira Santos J, Vila Real M, Santos F. Autoimmune encephalitis: the clinical evolution as a key to the diagnosis. BMJ Case Rep. 2019;12(9).

275. Terada A, Tasaki S, Tachibana T, Sakamoto Y, Yokomine M, Shimomura T, et al. Two cases of acute limbic encephalitis in which symptoms improved as a result of laparoscopic salpingo-oophorectomy. Gynecol Minim Invasive Ther. 2017;6(1):34-7.

276. Thomas A, Rauschkolb P, Gresa-Arribas N, Schned A, Dalmau JO, Fadul CE. Anti-N-methyl-D-aspartate receptor encephalitis: a patient with refractory illness after 25 months of intensive immunotherapy. JAMA Neurol. 2013;70(12):1566-8.

277. Thompson DC, Bailey MR, Bowley D, Jacob S. Encephalitis on deployment in Kenya: think beyond the infections. J R Army Med Corps. 2019;165(5):374-6.

278. Tokunaga S, Ide M, Ishihara T, Matsumoto T, Maihara T, Kato T. Transient extreme spindles in a young child with anti-NMDAR encephalitis: A case report. Brain Dev. 2019;41(2):210-3.

279. Tu NP, Nha PB, Hung ND, Minh NH, Anh HN, Dinh TC. Treatment of Anti-NMDA Receptor Encephalitis with Ovarian Teratoma Removal: A Literature Review and Two Case Reports. Open Access Maced J Med Sci. 2019;7(24):4378-82.

280. Turnbull MT, Siegel JL, Becker TL, Stephens AJ, Lopez-Chiriboga AS, Freeman WD. Early Bortezomib Therapy for Refractory Anti-NMDA Receptor Encephalitis. Front Neurol. 2020;11:188.

281. Uchida Y, Kato D, Yamashita Y, Ozaki Y, Matsukawa N. Failure to improve after ovarian resection could be a marker of recurrent ovarian teratoma in anti-NMDAR encephalitis: a case report. Neuropsychiatr Dis Treat. 2018;14:339-42.

282. Uchino A, Iizuka T, Urano Y, Arai M, Hara A, Hamada J, et al. Pseudo-piano playing motions and nocturnal hypoventilation in anti-NMDA receptor encephalitis: response to prompt tumor removal and immunotherapy. Intern Med. 2011;50(6):627-30.

283. Udani V, Desai N, Botre A. Partial Manifestation of Anti-NMDA-R Encephalitis with Predominant Movement Disorder. Mov Disord Clin Pract. 2016;3(1):80-2.

284. Valle DAD, Santos MLSF, Zeny MS, Cordeiro ML. Pediatric Anti-N-methyl-D-aspartate Receptor Encephalitis Mimicking Glutaric Aciduria Type 1: A Case Report. Frontiers in Neurology. 2020;11.

285. Van De Riet EHCW, Esseveld MM, Cuypers L, Schieveld JNM. Anti-NMDAR encephalitis: A new, severe and challenging enduring entity. European Child and Adolescent Psychiatry. 2013;22(5):319-23.

286. van der Meulen AAE, van der Hoeven JH, de Jong BM, Elting JWJ. Extreme delta brushes in anti NMDA receptor encephalitis - Muscle artefact or an EEG phenomenon? A case report. Clin Neurophysiol. 2017;128(10):1835-6.

287. Van Putten WK, Hachimi-Idrissi S, Jansen A, Van Gorp V, Huyghens L. Uncommon cause of psychotic behavior in a 9-year-old girl: a case report. Case Rep Med. 2012;2012:358520.

288. Vaux A, Robinson K, Saglam B, Cheuk N, Kilpatrick T, Evans A, et al. Autoimmune Encephalitis in Long-Standing Schizophrenia: A Case Report. Front Neurol. 2021;12:810926.

289. Voice J, Ponterio JM, Lakhi N. Psychosis secondary to an incidental teratoma: a "heads-up" for psychiatrists and gynecologists. Arch Womens Ment Health. 2017;20(5):703-7.

290. Vural A, Arsava EM, Dericioglu N, Topcuoglu MA. Central neurogenic hyperventilation in anti-NMDA receptor encephalitis. Intern Med. 2012;51(19):2789-92.

291. Wada N, Tashima K, Motoyasu A, Nakazawa H, Tokumine J, Chinzei M, et al. Anesthesia for patient with anti-N-methyl-Daspartate receptor encephalitis: A case report with a brief review of the literature. Medicine (United States). 2018;97(50).

292. Wali SM, Cai A, Rossor AM, Clough C. Appearance of anti-NMDAR antibodies after plasma exchange and total removal of malignant ovarian teratoma in a patient with paraneoplastic limbic encephalopathy. BMJ Case Rep. 2011;2011.

293. Walker CA, Poulik J, D'Mello RJ. Anti-NMDA receptor encephalitis in an adolescent with a cryptic ovarian teratoma. BMJ Case Rep. 2021;14(7).

294. Wallengren S, Johansson BA, Rask O. Acute manic state with psychotic features in a teenager with autoimmune encephalitis: a case report. J Med Case Rep. 2021;15(1):295.

295. Wang B, Wang C, Ren H, Guan H, Guo S. Cerebellar ataxia as the initial symptom with lesions involving the cerebellum in patient with anti-NMDAR encephalitis: A rare case report and literature review. J Neuroimmunol. 2020;346:577293.

296. Wang HY, Li T, Li XL, Zhang XX, Yan ZR, Xu Y. Anti-N-methyl-D-aspartate receptor encephalitis mimics neuroleptic malignant syndrome: case report and literature review. Neuropsychiatr Dis Treat. 2019;15:773-8.

297. Weaver M, Griffey RT. Anti-N-Methyl-d-Aspartate Receptor Encephalitis as an Unusual Cause of Altered Mental Status in the Emergency Department. J Emerg Med. 2016;51(2):136-9.

298. Wilson JE, Shuster J, Fuchs C. Anti-NMDA receptor encephalitis in a 14-year-old female presenting as malignant catatonia: medical and psychiatric approach to treatment. Psychosomatics. 2013;54(6):585-9.

299. Wu GF, Mao B, Hu JS. Anti-N-methyl-D-aspartate receptor encephalitis: A case report of an over 2-year-old girl. Chinese Journal of Contemporary Pediatrics. 2014;16(4):432-4.

300. Xu L, Chen Z. Anti-NMDA Receptor Encephalitis Misdiagnosed As Generalized Anxiety Disorder: A Case Report. Cureus. 2021;13(12):e20529.

301. Xue CY, Dong H, Yang HX, Jiang YW, Yin L. Four-year-old anti-N-methyl-D-aspartate receptor encephalitis patient with ovarian teratoma: A case report. World J Clin Cases. 2021;9(19):5319-24.

302. Yan B, Wang Y, Zhang Y, Lou W. Teratoma-associated anti-N-methyl-D-aspartate receptor encephalitis: A case report and literature review. Medicine (Baltimore). 2019;98(21):e15765.

303. Yang P, Li L, Xia S, Zhou B, Zhu Y, Zhou G, et al. Effect of Clozapine on Anti-N-Methyl-D-Aspartate Receptor Encephalitis With Psychiatric Symptoms: A Series of Three Cases. Front Neurosci. 2019;13:315.

304. Yang XZ, Cui LY, Ren HT, Qu T, Guan HZ. Anti-NMDAR encephalitis after resection of melanocytic nevi: report of two cases. BMC Neurol. 2015;15:165.

305. Yang Y, Zhang B, Li M, Li J. Successful treatment with immunoadsorption therapy in four patients with severe and refractory anti-N-methyl-D-aspartate receptor encephalitis. J Clin Apher. 2021;36(6):886-92.

306. Yeshokumar AK, Sun LR, Klein JL, Baranano KW, Pardo CA. Gait Disturbance as the Presenting Symptom in Young Children With Anti-NMDA Receptor Encephalitis. Pediatrics. 2016;138(3).

307. Yeum TS, Lee J, Park SY, Joen Y, Kim BN. Childhood Onset of Anti-N-Methyl-D-Aspartate Receptor Encephalitis Without Teratoma Masquerading as a Psychotic Disorder. Soa Chongsonyon Chongsin Uihak. 2019;30(3):127-31.

308. Yin H, Zhu C, Ren H, Yang X, Peng B, Cui L, et al. Resection of melanocytic nevi as a potential treatment of anti-NMDAR encephalitis patients without tumor: report of three cases. Neurol Sci. 2018;39(1):165-7.

309. Yoga B, Kunc M, Ahmed F. A case of non-paraneoplastic anti-N-methyl d-aspartate receptor encephalitis presenting as a neuropsychiatric disorder. SAGE Open Med Case Rep. 2014;2:2050313x14558902.

310. Yoshimura B, Yada Y, Horigome T, Kishi Y. Anti-N-Methyl-D-Aspartate Receptor Encephalitis Presenting With Intermittent Catatonia. Psychosomatics. 2015;56(3):313-5.

311. Yu M, Li S, Cheng J, Zhou L, Jiang Z, Di W. Ovarian teratoma-associated anti-NMDAR encephalitis: a single-institute series of six patients from China. Arch Gynecol Obstet. 2021;303(5):1283-94.

312. Yuan N, Glezer A. A young woman presenting with psychotic and mood symptoms from anti-N-methyl-D-aspartate receptor (NMDA-R) encephalitis: an emerging diagnosis. Int J Psychiatry Med. 2013;46(4):407-15.

313. Zheng F, Ye X, Shi X, Poonit ND, Lin Z. Management of Refractory Orofacial Dyskinesia Caused by Anti-N-methyl-d-aspartate Receptor Encephalitis Using Botulinum Toxin. Front Neurol. 2018;9:81.
